# ATF3-CBS signaling axis coordinates ferroptosis and tumorigenesis in colorectal cancer

**DOI:** 10.1016/j.redox.2024.103118

**Published:** 2024-03-08

**Authors:** Junjia Liu, Xinyi Lu, Siyu Zeng, Rong Fu, Xindong Wang, Lingtao Luo, Ting Huang, Xusheng Deng, Hualei Zheng, Shaoqian Ma, Dan Ning, Lili Zong, Shu-Hai Lin, Yongyou Zhang

**Affiliations:** a State Key Laboratory of Cellular Stress Biology, Innovation Center for Cell Signaling Network, Engineering Research Centre of Molecular Diagnostics of the Ministry of Education, School of Life Sciences, Xiamen University, Xiamen, Fujian, 361102, China; b National Institute for Data Science in Health and Medicine Engineering, Faculty of Medicine and Life Sciences, Xiamen University, Xiamen, Fujian, 361102, China; c School of Pharmaceutical Sciences, Xiamen University, Xiamen, Fujian, 361102, China; d Department of Gastrointestinal Surgery, The First Affiliated Hospital of Xiamen University, Xiamen University, Xiamen, Fujian, 361102, China

**Keywords:** Ferroptosis, Colorectal cancer (CRC), ATF3-CBS signaling axis, Mitochondrial tricarboxylic acid (TCA) cycle

## Abstract

The induction of ferroptosis is promising for cancer therapy. However, the mechanisms enabling cancer cells to evade ferroptosis, particularly in low-cystine environments, remain elusive. Our study delves into the intricate regulatory mechanisms of Activating transcription factor 3 (ATF3) on Cystathionine β-synthase (CBS) under cystine deprivation stress, conferring resistance to ferroptosis in colorectal cancer (CRC) cells. Additionally, our findings establish a positively correlation between this signaling axis and CRC progression, suggesting its potential as a therapeutic target. Mechanistically, ATF3 positively regulates CBS to resist ferroptosis under cystine deprivation stress. In contrast, the suppression of CBS sensitizes CRC cells to ferroptosis through targeting the mitochondrial tricarboxylic acid (TCA) cycle. Notably, our study highlights that the ATF3-CBS signaling axis enhances ferroptosis-based CRC cancer therapy. Collectively, the findings reveal that the ATF3-CBS signaling axis is the primary feedback pathway in ferroptosis, and blocking this axis could be a potential therapeutic approach for colorectal cancer.

## Highlights

• Cystine deprivation activates ATF3-CBS signaling axis.
• ATF3-CBS signaling axis positively correlates with CRC progression.
• Suppression of CBS sensitizes CRC cells to ferroptosis by targeting the mitochondrial TCA cycle.
• Ablation of CBS enhances ferroptosis-based cancer therapies for CRC.

## Graphical abstract

**Figure:**
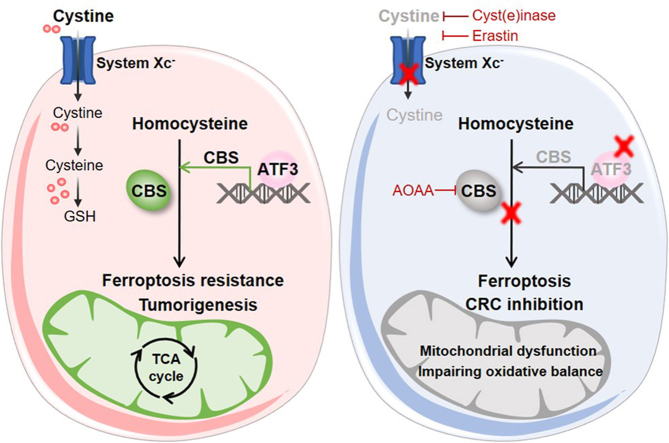
The substitution of Eu ion enhanced the magnetic and dielectric properties of Co-Zn-Ni ferrites significantly by the solid-state reaction method.

## 1 Introduction

Tumor cells use multiple remodeled synthetic and catabolic pathways to support their survival and proliferation in nutrient-poor microenvironments [1]. One such pathway, amino acid metabolism, is essential for cancer cells to overcome nutrient deprivation and oxidative stress [2]. Previous studies have indicated that sulfur amino acid restriction inhibits tumor angiogenesis [3] and disrupts intracellular redox homeostasis [4]. Furthermore, the restriction of amino acids, such as methionine, inhibits tumor growth in patient-derived xenograft (PDX) models of colorectal cancer (CRC) [5] and induces ferroptosis in tumor cells [[6], [7], [8]]. Therefore, amino acid restriction has been extensively studied as a strategy for efficient cancer therapy [[10], [11], [9]].

Cysteine is a non-essential amino acid required for cell proliferation and glutathione (GSH) synthesis. In addition to acquiring exogenous cystine via system Xc^−^, mammalian cells synthesize cysteine by converting methionine to cysteine via the reverse transsulfuration pathway [12,13] in which homocysteine is synthesized from methionine through the methionine cycle. Cystathionine β-synthase (CBS) catalyzes the synthesis of cystathionine from homocysteine and serine. Cystathionine is then converted to cysteine by cystathionine γ-lyase (CTH) [14]. Since the tumor microenvironment contains low levels of glutamine and cysteine, it is difficult for tumor cells to maintain the optimum level of cysteine solely by utilizing system Xc^−^ [15]. Thus, tumor cells use the transsulfuration pathway-mediated cysteine biosynthesis to support growth in the cysteine-limited tumor microenvironment, which is required for cancer initiation and progression [16]. Despite the central role of CBS in the transsulfuration pathway and metabolism of sulfur-containing amino acids under physiological conditions [17], the regulation of CBS in cancer cells, which are under ferroptotic stress due to nutrient deprivation, remains unknown.

Ferroptosis, an increasingly studied form of cell death driven by the accumulation of iron-dependent lipid peroxides on the cell membrane [18], has recently been shown to be important for tumor progression [19]. Although the physiological function of ferroptosis remains elusive, there is increasing evidence that its induction is a promising therapeutic strategy for various cancers [20]. One way to induce ferroptosis is to limit the transport of exogenous cystine [6] by blocking the transmembrane amino acid transporter system Xc^−^ composed of SLC7A11 (XCT) and SLC3A2 [21], or by depleting cystine via cyst(e)inase-mediated degradation [22]. Internalized cystine is converted to cysteine and generating GSH [23]. Inhibition of GSH synthesis is sufficient to stimulate ferroptosis in most cells [24], as GSH is a tripeptide antioxidant that acts as a cofactor of selenium-dependent GPX4 to reduce lipid hydroperoxides [25]. Therefore, ferroptosis inducers, such as Erastin, an inhibitor of the cystine/glutamate antiporter system Xc^−^, have attracted considerable interest in cancer-targeted therapies [24,26]. Additionally, cysteine depletion by cyst(e)inase induces ferroptosis in pancreatic tumor cells in mice [6,22]. However, details regarding upstream and downstream mediators and potential clinical approaches targeting these mediators remain elusive.

To meet the growing demand for biomass accumulation, energy production, and maintenance of redox homeostasis, the metabolism of tumor cells has undergone profound changes in response to nutrients such as amino acids [27]. Primary integrated stress response (ISR) sensors, including ElF2α, ATF3, ATF4 and GCN2 are important regulators that respond to stress induced by amino acid deprivation [3,28]. Therefore, amino acid deprivation and regulation of ISR stress response are attractive targets for cancer therapy [29,30]. In this study, we systematically investigated the mechanism of CBS regulation during ferroptosis and demonstrated that the ATF3-CBS signaling axis is a potential therapeutic target for CRC.

## 2 Results

### 2.1 Suppression of CBS promotes ferroptosis under cystine-restricted conditions

**Fig. 1:**
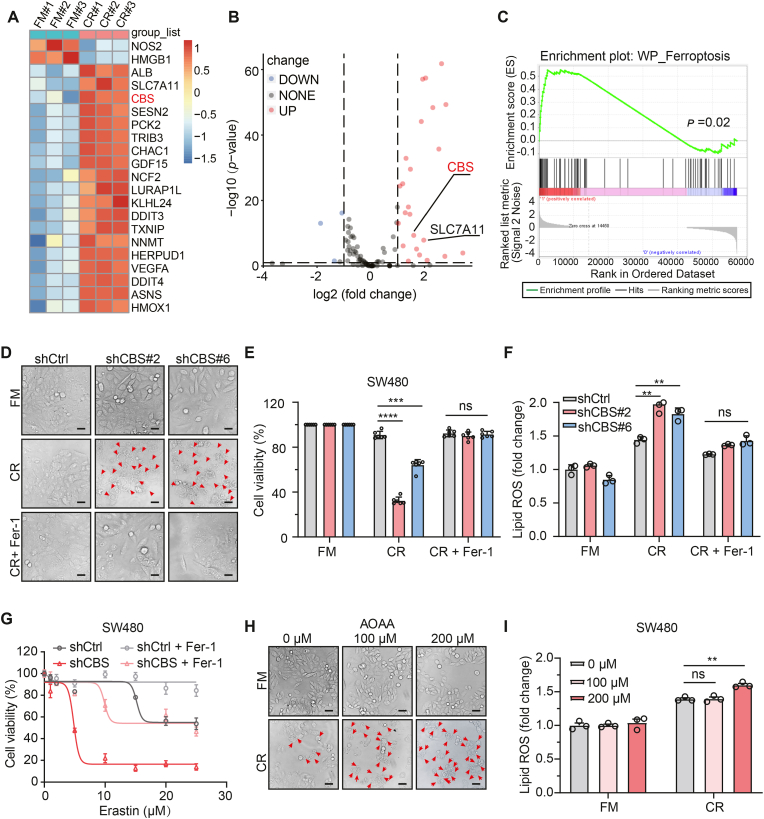
Suppression of CBS promotes ferroptosis under cystine-restricted conditions. **A.** Heatmap of normalized gene expression (z score) determined by RNA-seq analysis of SW480 cell lines cultured in the full medium (FM) or cystine-restricted (CR) medium for 36 h. For comparing cells cultured in CR medium to those cultured in complete medium, red indicates upregulated genes, while blue indicates downregulated genes. **B.** Volcano plot showing downregulated genes (blue) and upregulated genes (red) in SW480 cells cultured with FM compared with CR medium. **C.** GSEA of CBS-related genes based on RNA-seq data for the control and CBS KD groups of SW480 cells. **D.** Micrographs of control and CBS KD SW480 cells cultured under cystine restriction alone or in combination with 2 μM Fer-1 treatment for 24 h. The red arrows indicate ferroptotic cells. Scale bar, 50 μm **E.** The viability of SW480 cells cultured under cystine restriction for 24 h alone or in combination with Fer-1 as indicated is shown. **F.** Lipid ROS were quantified in CBS KD SW480 cells after cystine starvation alone or in combination with 2 μM Fer-1 treatment for 24 h by C11-BODIPY staining and flow cytometric analysis. **G**. Viability of SW480 cells treated with Erastin alone or in combination with 2 μM Fer-1. Student's *t*-test was used to compare the maximal cytotoxicity with and without Fer-1. **H.** Micrographs of SW480 cells treated with 0, 100 or 200 μM AOAA alone or in combination with cystine restriction for 24 h. The red arrows indicate ferroptotic cells. Scale bar, 50 μm. **I.** Lipid ROS production was quantified by C11-BODIPY staining and flow cytometric analysis. Data are presented as mean ± SEM, and **p < 0.01, ***p < 0.001, ****p < 0.0001; “ns” indicates not significant compared to control group or the indicated two groups, based on two-tailed, unpaired Student's *t*-test or Pearson *r*-test.

We compared the whole-genome transcription profiles of the SW480 colorectal cancer (CRC) cell line cultured in full medium (FM) and cystine-restricted (CR) medium to identify changes in gene expression in response to cystine deprivation. *CBS* and *SLC7A11* gene expression was substantially upregulated after incubation in CR medium for 36 h (Fig. 1A and B), consistent with a previous study showing that cystathionine β-synthase (CBS) and the system Xc^−^ amino acid antiporter SLC7A11 are essential for cysteine metabolism and ferroptosis induced by cystine depletion [16]. Moreover, gene set enrichment analysis (GSEA) showed that genes upregulated in response to cystine deprivation are enriched in the ferroptotic signature with CBS knockdown (KD) CRC cells (Fig. 1C). Together, these data demonstrated that CBS is linked to ferroptosis caused by cystine restriction.

To explore the role of CBS in ferroptosis, four CRC cell lines were used to test their sensitivity to ferroptosis induced by cystine deprivation or Erastin treatment. Interestingly, SW480 and SW620 cells with high CBS expression were temporarily resistant, although they expressed similar levels of SLC7A11 (Figs. S1A–C). We further established stable CBS KD cell lines by using two specific short hairpin RNAs (shCBS#2 and shCBS#6) (Figs. S1D and E). Knockdown of CBS in all tested CRC cell lines increased ferroptosis induced by cystine restriction (Fig. 1D–F and Figs. S1F–H) and Erastin treatment (Fig. 1G, Figs. S1I and J), which was confirmed by decreased cell viability and increased accumulation of lipid peroxidation (lipid ROS; a biomarker of ferroptosis that the fluorescent probe C11-BODIPY can detect). In addition, treatment with ferrostatin-1 (Fer-1, a specific ferroptosis inhibitor) markedly reduced cell death (Fig. 1D, E and Fig. 1G) and lipid ROS accumulation in control and CBS KD cells under CR medium (Fig. 1F and Figs. S2A–C).

Similar to the effect of CBS KD, treatment with Aminooxyacetic acid (AOAA; a common inhibitor of CBS) in combination with CR also enhanced ferroptosis (Fig. 1H, I and Figs. S2D–G). Additionally, CBS overexpression in MEFs inhibited ferroptosis (Figs. S2H–K). Collectively, the data indicate that suppression of CBS sensitizes CRC cells to ferroptosis under cystine-restricted conditions.

### 2.2 Depletion of CBS enhances ferroptosis-based cancer therapies for CRC

**Fig. 2:**
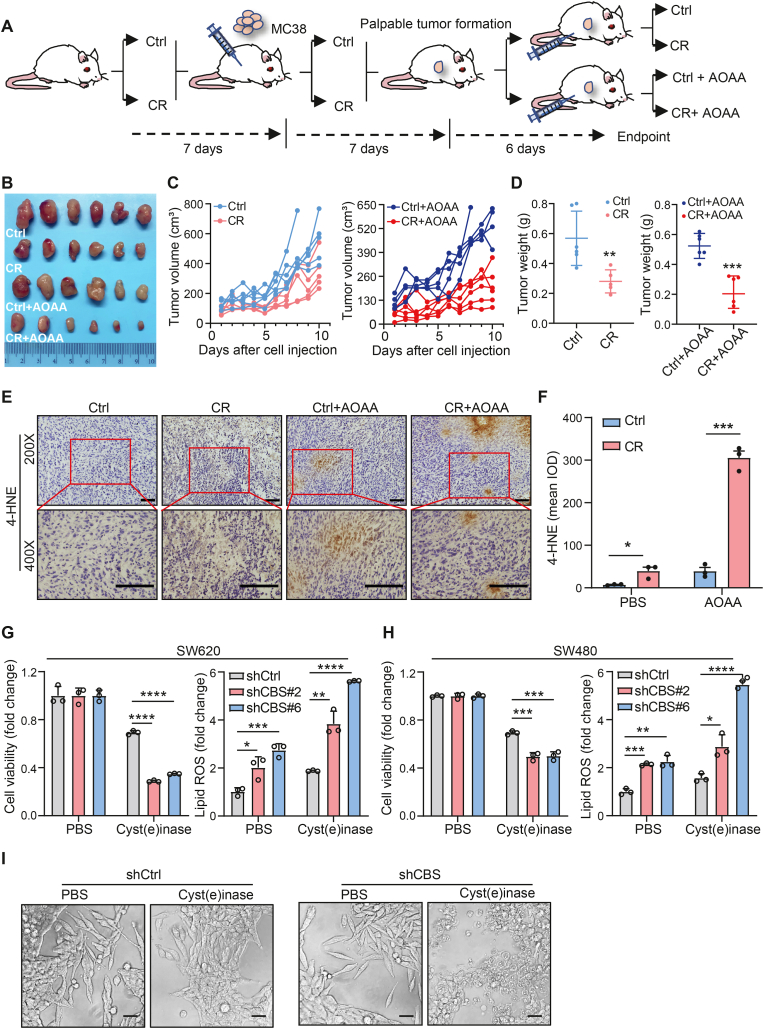
Depletion of CBS enhances ferroptosis-based cancer therapies for CRC. **A.** Schematic of the experimental design for the intraperitoneal injection of MC38 cells to establish a xenograft model. The control diet (FM) or cystine-restricted diet (CR) or received CBS inhibitor, AOAA (i.P.) treatment of C57BL/6 mice, AOAA = 9 mg/kg, n = 6 mice per group. **B.** Representative images of tumors at the endpoint. **C** and **D.** Tumor growth curves in mice on dietary treatment alone or in combination with AOAA treatment. The final masses of the subcutaneous tumors were determined. Treatment, n = 6 mice per group. **E.** 4-HNE staining of tumors. Scale bar, 100 μm. Magnified view at 400 × of the area highlighted at 200 × by the red squares is depicted in the bottom row. Scale bar, 100 μm **F.** Quantification of 4-HNE-positive cells in tumors. (n = 3 tumors). **G** and **H.** Cell viability and lipid ROS production were quantified of CBS KD in SW620 and SW480 cells after cyst(e)inase treatment for 24 h. **I.** Micrographs of control and CBS KD SW620 cells treated with cyst(e)inase for 24 h. Scale bar, 100 μm. Data are presented as mean ± SEM., and *p < 0.05, **p < 0.01, ***p < 0.001, ****p < 0.0001 compared to control group or the indicated two groups, based on two-tailed, unpaired Student's *t*-test.

To investigate the potential of a cystine-restricted diet combined with inhibition of CBS in the prevention or treatment of CRC, eight-week-old C57BL/6 mice were subjected to either a standard control diet or a cystine-restricted diet. The dietary intervention commenced one week prior to initiating xenograft experiments with mouse CRC cells (MC38), and it was consistently maintained throughout the entire experimental period. Additionally, one group of mice also received an intraperitoneal injection of the CBS inhibitor, AOAA (Fig. 2A). Subsequently, the cystine-restricted diet markedly reduced tumor growth, and this reduction was more pronounced in the group treated in combination with AOAA (Fig. 2B–D), indicating that depletion of CBS and cystine deprivation synergistically inhibits CRC tumorigenesis. Moreover, immunohistochemical (IHC) staining showed that tumors in mice fed the cystine-restricted diet and treated with AOAA exhibited substantial accumulation of 4-hydroxynonenal (4-HNE), a byproduct of lipid peroxidation indicating ferroptosis in vivo (Fig. 2E and F). These data indicate that CBS depletion promotes cystine restriction-induced ferroptosis in CRC; thus, dietary cystine restriction combined with CBS depletion shows therapeutic promise in CRC.

Recombinant cyst(e)inase has been reported to deplete both cysteine and cystine in the tumor microenvironment. It is a safe and effective treatment for pancreatic tumors, prostate cancer, and breast cancer xenografts [22]. Indeed, in vitro, knockdown of CBS exacerbated the reduced cell viability and increased lipid peroxide accumulation induced by the addition of cyst(e)inase to the culture medium (Fig. 2G–I). Therefore, depletion of cyst(e)ine in the tumor microenvironment combined with inhibition of intracellular CBS could be a more potent therapy for CRC.

### 2.3 Suppression of CBS sensitizes CRC cells to ferroptosis by targeting the mitochondrial TCA cycle

**Fig. 3:**
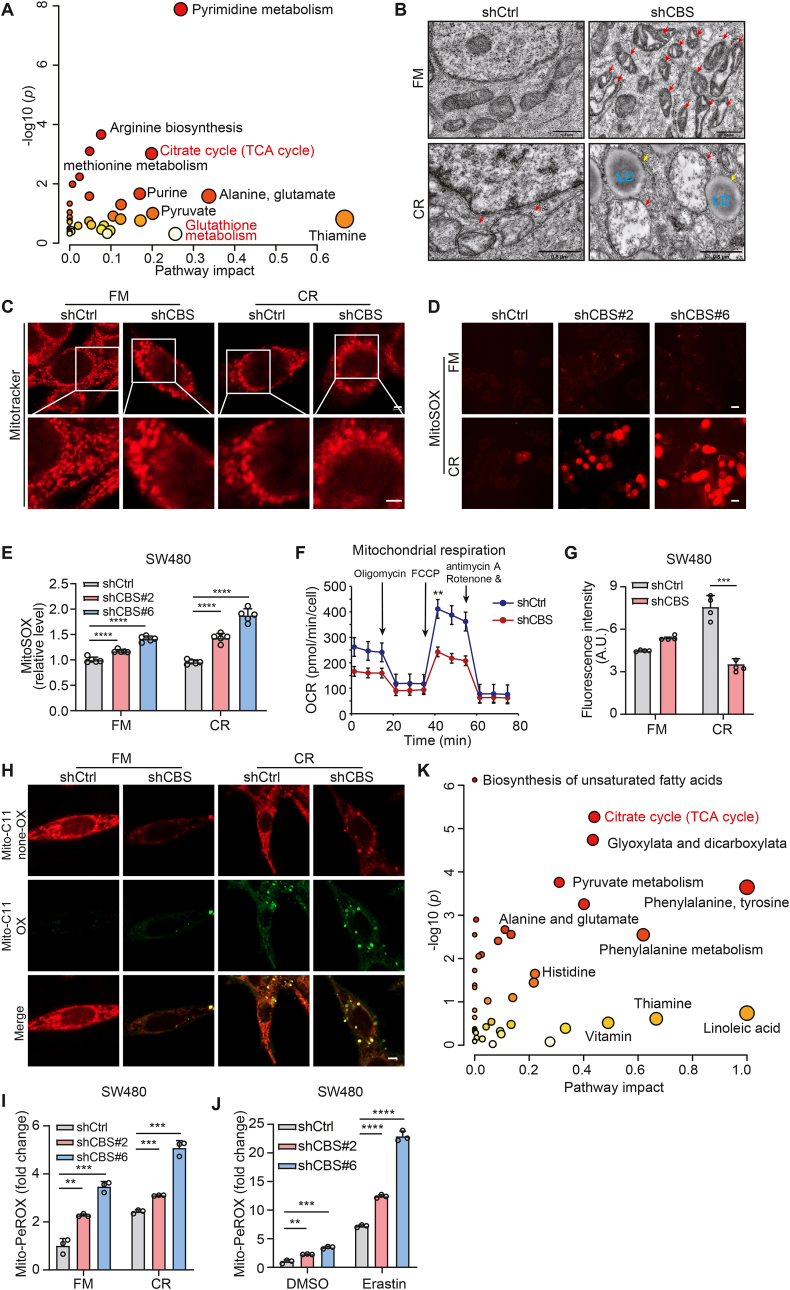
Suppression of CBS sensitizes CRC cells to ferroptosis by targeting the mitochondrial TCA cycle. **A.** Metabolite sets showed differential abundance between CBS KD cells and control cells. The metabolites with significantly different abundance between the two groups of cells were subjected to pathway enrichment analysis (y-axis, enrichment *p* values) and pathway topology analysis (x-axis, pathway impact values, indicative of the centrality and enrichment of a pathway) (n = 5 replicates per group, *p* < 0.05, |FC|>1.5). Circle color indicates the level of enrichment significance, with yellow indicating low significance and red indicating high significance. Circle size is proportional to the impact value of the pathway. **B.** TEM images of SW480 cells in the control and CBS KD groups cultured under cystine restriction for 12 h. The red arrowheads indicate mitochondria, and the yellow arrowheads indicate lipid droplets (LD); scale bar, 0.5 μm **C.** Mitochondrial morphology was determined by Mitotracker Red staining and confocal imaging. Scale bar, 2 μm. Magnified view of the region indicated by the white squares are shown on the bottom row. Scale bar, 2 μm. **D.** MitoSOX staining of live SW480 cells undergoing cystine restriction-induced ferroptosis. Scale bar, 50 μm **E.** Mitochondrial superoxide production was quantified in SW480 cells after cystine starvation for 24 h by MitoSOX staining and flow cytometric analysis. **F.** OCR in the control and CBS KD SW480 groups. The results of a representative experiment with n = 5 technical replicates per treatment are shown. **G.** The cellular ATP levels were measured in SW480 cells under cystine restriction for 24 h and normalized to the number of cells (n = 4 technical replicates per treatment). **H.** The control and CBS KD SW480 cells were treated with cystine restriction for 12 h, then stained with mito-BODIPY. Oxidized mito-BODIPY (green) indicates mitochondrial lipid peroxidation. Scale bar, 5 μm. **I** and **J.** Mitochondrial lipid peroxidation production was quantified in SW480 cells after cystine restriction or Erastin treatment for 24 h by MitoPerOX staining and flow cytometric analysis. **K.** Metabolites of Mitochondria sets showed differential abundance between CBS KD cells and control cells. Data are presented as mean ± SEM., and **p < 0.01, ***p < 0.001, ****p < 0.0001 compared to control group or the indicated two groups, based on two-tailed, unpaired Student's *t*-test.

We further explored the mechanism of CBS in ferroptosis of CRC cells. Considering that CBS is a key metabolic enzyme in the transsulfuration pathway (Fig. S3A), we conducted an unbiased LC-MS-based large-scale targeted metabolomic analysis. Differences in the abundance of 154 metabolites between the control and shCBS SW480 cells were revealed, with enrichment in glutathione (GSH) and mitochondrial tricarboxylic acid (TCA) cycle pathway associated with ferroptosis (Fig. 3A and Figs. S3B and C). Indeed, CBS depletion had no effect on the total abundance of GSH or the GSH:GSSG ratio in SW480 cells (Fig. S3D). Furthermore, supplementation of cysteine or glutathione, the downstream metabolites of the transsulfuration pathway, only partially alleviated CBS KD-induced ferroptosis (Figs. S3E-L).

Since the mitochondrial TCA cycle promotes cystine deprivation-induced ferroptosis [10], we further explored whether CBS regulated ferroptosis via mitochondria. Subsequently, we monitored the morphological changes of mitochondria, a key ferroptosis feature, using transmission electron microscopy (TEM). TEM revealed heightened mitochondrial stress in CBS KD cells, manifested by swollen mitochondrial and structural aberrations in mitochondrial. Under cystine restriction, mitochondrial in CBS KD cells exhibited cristae disruption and larger lipid droplets compared to control cells (Fig. 3B and Fig. S4A). Next, mitochondrial morphology alterations were visualized through MitoTracker Red labeling. In contrast to the long and filamentous morphology observed in the control group, CBS KD cells predominantly displayed spherical and circular mitochondrial morphology (Fig. 3C), indicating mitochondrial fusion and fission due to CBS suppression. Moreover, mitochondrial and intracellular ROS levels were significantly increased in CBS KD cells under cystine restriction, as indicated by MitoSOX and DCFH-DA staining (Fig. 3D, E and Figs. S4B, C). To determine the impact of CBS deficiency on mitochondria function, we further assessed changes in mitochondrial basal metabolism. A reduction in the oxygen consumption rate (OCR) was observed in CBS KD cells (Fig. 3F). Correspondingly, CBS KD cells showed diminished ATP levels in individual cells (Fig. 3G), indicating mitochondria function was impaired. Moreover, the knockdown of CBS increased mitochondrial lipid peroxidation, as indicated by mito-BoDIPY (Fig. 3H–J). We further detected differences in mitochondrial metabolites by an anti-HA IP utilizing the control and shCBS SW480 cells expressing the 3 × HA-EGFP-OMP25 (HA-MITO) (Fig. S4D). Differential analysis revealed that 91 mitochondrial metabolites between control cells and shCBS SW480 cells were enriched in pathways related to the TCA cycle (Fig. 3K and Fig. S4E). Overall, these results demonstrate that CBS suppression in CRC cells disrupts mitochondrial by targeting the mitochondrial TCA cycle.

### 2.4 ATF3-activated CBS protects CRC cells from ferroptosis

**Fig. 4:**
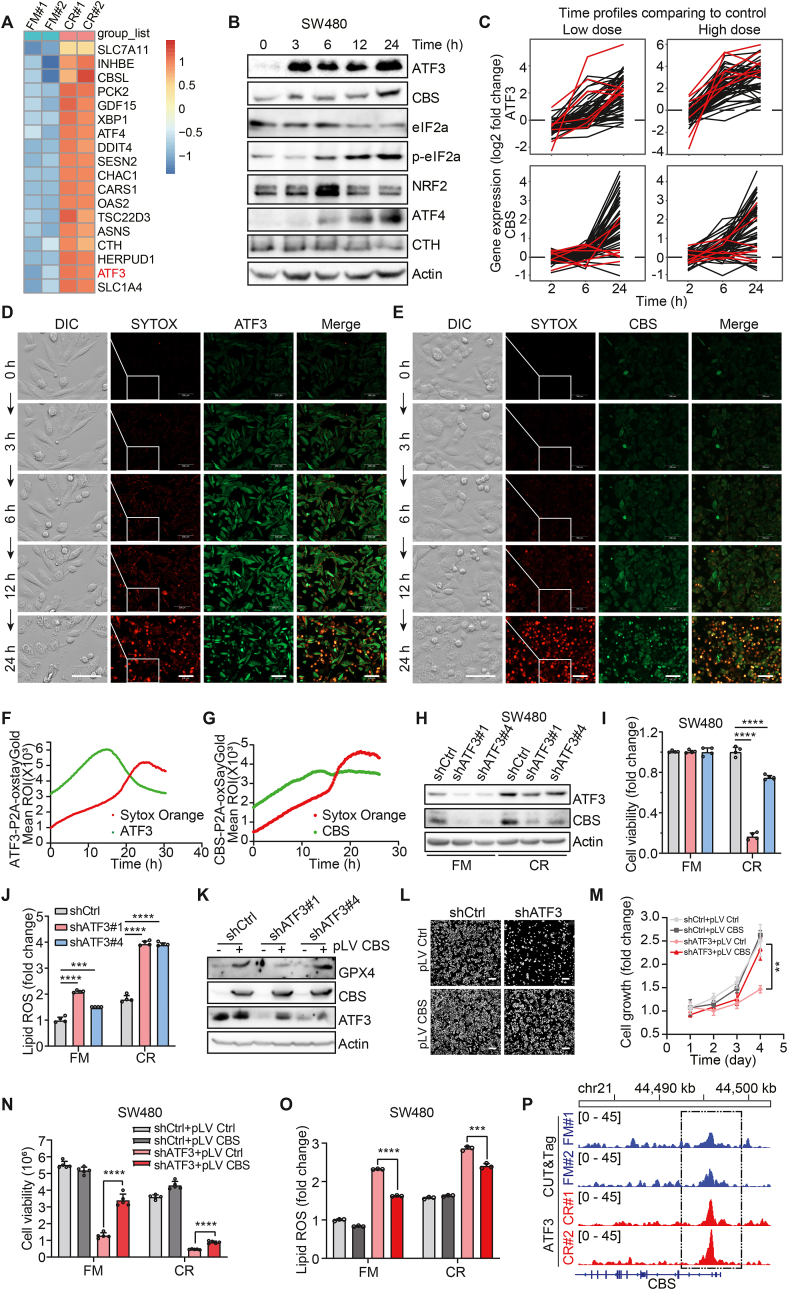
ATF3-activated CBS protects CRC cells from ferroptosis. **A.** Heatmap of normalized gene expression (z score) based on RNA-seq data for SW480 cells cultured in CR medium or FM for 6 h. **B.** Western blot analysis of the changes of ISR-related proteins along cystine restriction. Relative protein expression was normalized to β-actin. **C.** The change in ATF3 and CBS expression in response to treatment with sorafenib at two different concentrations over three time periods (data from the NCI TPW online resource). **D** and **E.** Representative images from the photographed living SW480 cells. DIC, SYTOX Orange channels and ATF3-P2A-oxStayGold (**D**) or CBS-P2A-oxStayGold (**E**) in living SW480 cells were photographed by the fully automatic live cell microscopy imaging system with the prolongation of cystine-limited treatment time. SYTOX Orange-positive cells are dead. Scale bar in fluorescence channel, 100 μm. Detailed depiction of cell morphology in the area highlighted by the white squares is presented in the left column (DIC). Scale bar in DIC, 50 μm. **F** and **G.** Real-time fluorescence change curves of ATF3-P2A-oxStayGold (**F**) or CBS-P2A-oxStayGold (**G**) and SYTOX Orange channels in living SW480 cells (**D** and **E**) under cystine restriction conditions. **H.** Western blot analysis of CBS in ATF3 KD SW480 cells under cystine restriction for 24 h. Relative protein expression was normalized to β-actin. **I.** Viability of control and ATF3 KD SW480 cells under cystine restriction or incubated in FM for 24 h. **J.** Lipid ROS production was quantified in ATF3 KD SW480 cells after cystine restriction or incubation in FM for 24 h by C11-BODIPY staining. **K.** Western blot analysis of ATF3, CBS and GPX4 (a classic marker of ferroptosis), in ATF3 KD SW480 cells with/without CBS overexpression after incubation in CR medium or FM for 24 h. Relative protein expression was normalized to β-actin. **L.** Representative images of cell density indicating cell proliferation in each group seeded with the same number of cells. ATF3 KD and control SW480 cells with or without CBS overexpression are shown. Scale bar, 200 μm. **M.** Curves showing the fold change in the growth of SW480 cells in the control group and ATF3 KD group with CBS overexpression. **N.** Viability of control and ATF3 KD SW480 cells with or without CBS overexpression after incubation in CR medium or FM for 24 h. **O.** Lipid ROS production was quantified in ATF3 KD and control SW480 cells with/without CBS overexpression after incubation in CR medium or FM for 24 h by C11-BODIPY staining and flow cytometric analysis. **P.** Genome Browser views of ATF3 CUT&Tag profiles at CBS loci in SW480 cells after cystine restriction for 24 h or incubation in FM as the control group. Data are presented as mean ± SEM, and **p < 0.01, ***p < 0.001, ****p < 0.0001 compared to control group or the indicated two groups, based on two-tailed, unpaired Student's *t*-test.

To identify the potential upstream transcription factors responsible for CBS induction during ferroptosis, we analyzed the expression of transcription factors related to ferroptosis. Subsequently, significant upregulation in ATF3 expression was discerned following a 6 h incubation in CR medium (Fig. 4A). Next, we evaluated the expression of proteins related to the integrated stress response (ISR) and the transsulfuration pathway of amino acid metabolism. ATF3 was activated within 3 h of CR treatment and CBS in the transsulfuration pathway was upregulated subsequently (Fig. 4B). To further determine the regulatory role of CBS and ATF3 in cancer cells response to ferroptosis, we analyzed data from the NCI Transcriptional Pharmacodynamics Workbench [31], which contains expression data for 12,704 genes in the NCI-60 panel of cell lines exposed to sorafenib, a candidate ferroptosis inducer [32]. ATF3 and CBS were among the most upregulated genes at 2, 6, and 24 h after sorafenib treatment (Fig. 4C). Consequently, these data suggested that ATF3 and CBS are related to ferroptosis.

A previous study showed that ferroptosis develops in a propagated manner [33]. To quantitatively study the propagation of ATF3-activated CBS induction and ferroptosis, we performed live-cell imaging of the SW480 cell line with endogenous ATF3 and CBS labeled with P2A-OxstayGold respectively in cystine restriction medium in the presence of the cell death indicator SYTOX Orange. We found that increased ATF3 expression occurred within 3 h, followed by increased CBS expression prior to ferroptosis and swelling morphology in the treated cells (Fig. 4D–G). Together, the upregulation of ATF3 and CBS can serve as an early hallmark event during cystine restriction-induced ferroptosis.

To explore the role of ATF3-CBS signaling axis in ferroptosis, we knocked down ATF3 in CRC cells and found that CBS expression is reduced in ATF3 KD cells (Fig. 4H and Fig. S5A), resulting in reduced cell proliferation (Figs. S5B and C) and increased ferroptosis (Fig. 4I and J, Figs. S5D–I). While ATF4 is a well-known transcription factor to amino acid stress. Surprisingly, ATF3 KD cells were more sensitive than ATF4 KD cells to cystine restriction-induced ferroptosis (Figs. S5J-L). This evidence led us to hypothesize that ATF3, rather than ATF4, functions as the primary transcriptional regulator of the response to cystine deprivation.

Moreover, CBS overexpression partially rescued ferroptosis induced by ATF3 KD (Fig. 4K-O and Fig. S6). To validate whether ATF3 directly regulates CBS expression at the transcriptional level, we performed Cleavage Under Targets and Tagmentation (CUT&Tag) to analyze the differences in ATF3 binding to regulatory elements between the FM and CR medium groups. ATF3 enrichment in the CBS promoter region substantially increased upon incubation in the CR medium (Fig. 4P). Together, these results demonstrate that ATF3 directly regulates the endogenous CBS expression, which responds to CR-induced ferroptotic stress.

### 2.5 Activation of the ATF3-CBS axis positively correlated with CRC progression

**Fig. 5:**
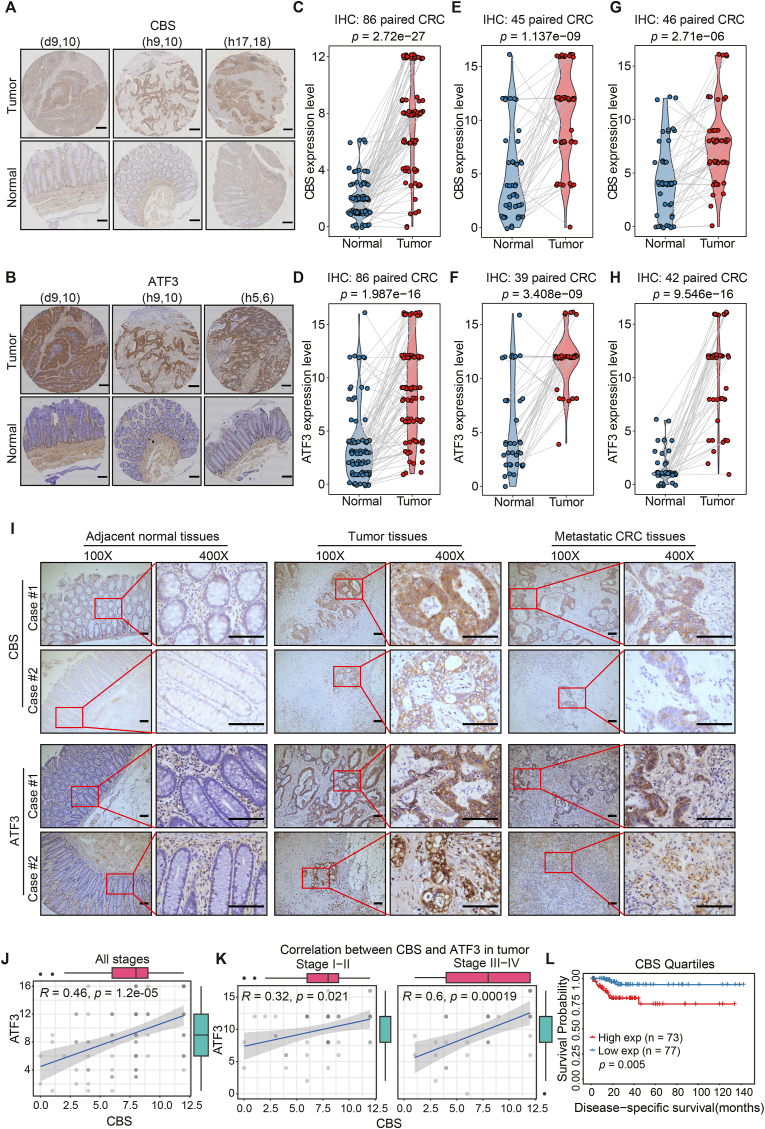
Activation of the ATF3-CBS axis positively correlated with CRC progression. **A.** Immunohistochemical staining of CBS in a tissue microarray containing samples from patients with colon cancer. Representative staining of CBS expression in representative primary CRC tissues and adjacent normal tissues (n = 86). (d9,10), (h9,10) and (h17,18) represent the coordinates in TMA (Fig. S7A). Scale bar, 200 μm **B.** IHC staining of ATF3 in a tissue microarray containing samples from patients with colon cancer. Staining of ATF3 in representative primary CRC tissues and adjacent normal tissues (n = 86). (d9,10), (h9,10) and (h5,6) represent the coordinates in TMA (Fig. S8A). Scale bar, 200 μm **C.** Paired correlation analysis following staining and quantification of CBS expression in representative primary CRC tissues and adjacent normal tissues (n = 86) in TMA. The scores are based on the intensity and extent (area) of staining (protein expression). **D.** Paired correlation analysis of staining and quantification of ATF3 expression in representative primary CRC tissues and adjacent normal tissues (n = 86) in TMA. **E.** Paired correlation analysis following staining and quantification of CBS expression in representative primary CRC tissues and adjacent normal tissues (n = 45) in TMA. **F.** Paired correlation analysis of staining and quantification of ATF3 expression in representative primary CRC tissues and adjacent normal tissues (n = 39) in TMA. **G.** Paired correlation analysis following staining and quantification of CBS expression in representative primary CRC tissues and adjacent normal tissues (n = 46). **H.** Paired correlation analysis of staining and quantification of ATF3 expression in representative primary CRC tissues and adjacent normal tissues (n = 42). **I.** Immunohistochemical staining of CBS/ATF3 in colon cancer patient samples. Representative staining of CBS/ATF3 in adjacent normal tissues, colon cancer foci and metastatic CRC foci. Scale bar, 100 μm **J** and **K.** Correlation between CBS and ATF3 by immunohistochemical analysis of gene expression in CRC TMA samples (n = 86). **L.** Kaplan-Meier analysis of disease-specific survival in a set of CRC patients according to CBS expression. Data are analyzed using paired *t*-test for (**C–H**); Pearson *r*-test (**I–K**) and Log rank test for (**L**).

After determining that ATF3 regulates CBS expression under ferroptotic stress conditions in CRC, we sought to confirm this finding in human CRC samples as a first step toward determining the potential value of targeting the ATF3-CBS signaling axis in CRC. The immunohistochemical (IHC) assay was used to detect the expression of CBS and ATF3 in a tissue microarray containing CRC samples from different stages and matched normal tissues (n = 86 pairs). ATF3 and CBS protein levels were significantly higher in CRC tissues than in the matched normal colon tissues (Fig. 5A–D, Figs. S7 and S8). Subsequently, the IHC assay of another tissue microarray (45 pairs to CBS and 39 pairs to ATF3) also confirmed the above results (Fig. 5E and F). To confirm this finding, an independent cohort of 46 paired CRC clinical samples with CBS and 42 paired with ATF3 were analyzed by IHC staining, which again revealed that CBS expression positively correlated with ATF3 expression in these CRC specimens (Fig. 5G–I, Figs. S9 and S10). Similarly, IHC staining showed that CBS and ATF3 were significantly higher in colorectal cancer hepatic metastasis samples than in adjacent normal tissues (Fig. 5I). Next, the Spearman correlation analysis indicated a significant correlation between the CBS and ATF3 expression in CRC tissues (Fig. 5J). Subsequently, we analyzed the IHC scores of CBS and ATF3 of CRC tissues in various clinical stages. The CBS and ATF3 expression in stages Ⅲ-Ⅳ were significantly higher than in stage Ⅰ-Ⅱ (Fig. 5K), indicating that CBS and ATF3 protein levels positively correlate with the clinical stage. Moreover, elevated CBS expression correlates with poorer patient survival (Fig. 5L and Fig. S11A). Thus, we conclude CBS and ATF3 are positively correlated with human CRC progression.

### 2.6 Depletion of CBS retards tumorigenesis in mouse CRC model

**Fig. 6:**
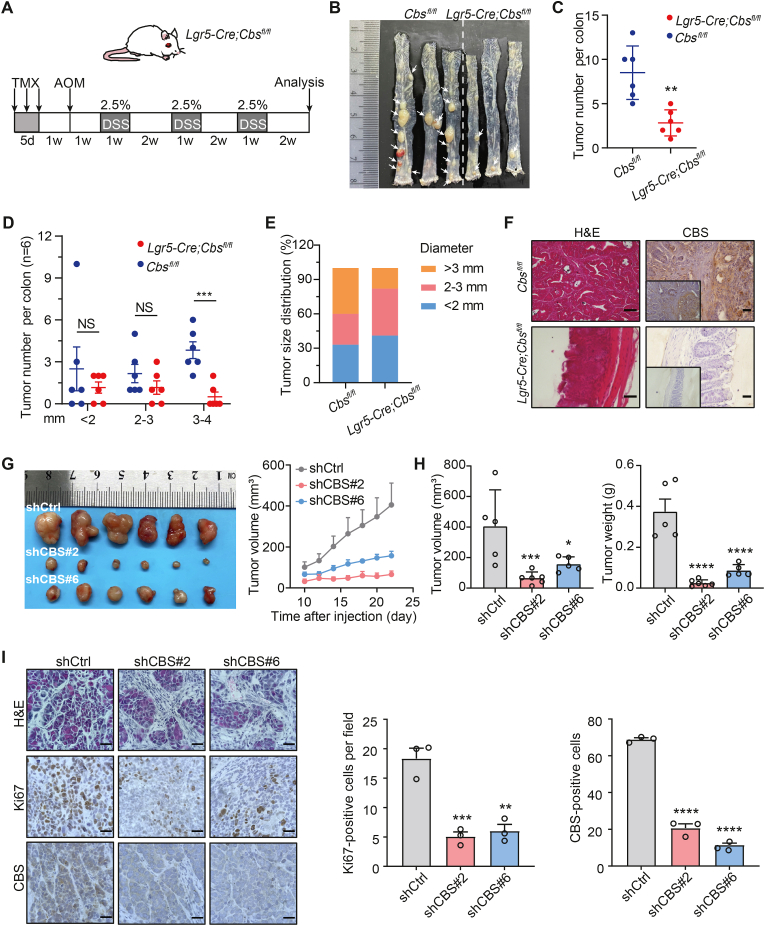
Depletion of CBS retards tumorigenesis in mouse CRC model. **A**. Schematic of the experimental design used to generate a mouse model of colitis-associated colon cancer. Azoxymethane (AOM) and DSS treatment of *Cbs*^*fl/fl*^ mice and *Lgr5-Cre*-*ERT2; Cbs*^*fl/fl*^ mice, n = 6 mice per group. **B**. Representative images of intact colon tumors in *Cbs*^*fl/fl*^ mice and *Lgr5-Cre ERT2; Cbs*^*fl/fl*^ mice on day 80 after injection of AOM. **C.** The total number of colon tumors in *Cbs*^*fl/fl*^ mice and *Lgr5-Cre*-*ERT2; Cbs*^*fl/fl*^ mice (n = 6). Each symbol represents an individual mouse. **D** and **E.** Analysis of the numbers of tumors of different sizes per colon (**G**) and percentage of tumors of different sizes (**H**) in *Cbs*^*fl/fl*^ mice and *Lgr5-Cre*-*ERT2; Cbs*^*fl/fl*^ mice (n = 6). Each symbol represents an individual mouse. **F.** H&E staining and IHC staining of CBS in CRC tissue sections collected from *Cbs*^*fl/fl*^ mice and *Lgr5-Cre*-*ERT2; Cbs*^*fl/fl*^ mice. Scale bar, 25 μm **G** and **H.** Tumor growth curves in immunodeficient nude mice injected subcutaneously with shCtrl or shCBS SW480 cells and monitored for 4 weeks (n = 6). The final volume and weight of the subcutaneous tumors were determined. **I.** H&E and IHC staining of Ki-67 and CBS in tissue sections from control and CBS KD SW480 subcutaneous tumors. Scale bar, 50 μm. Quantifying Ki-67- and CBS-positive cells in tumors (n = 3 tumors). Data are presented as mean ± SEM, and *p < 0.05, **p < 0.01, ***p < 0.001, ****p < 0.0001; “NS” indicates not significant compared to *Cbs*^*fl/fl*^ group or control group, based on two-tailed, unpaired Student's *t*-test.

Since ATF3 regulates multiple genes as a transcription factor, we investigated the potential of CBS as a clinical treatment target. We further explored whether CBS is involved in CRC tumorigenesis in vivo using a colitis-associated CRC mouse model. First, we crossed *Lgr5-Cre-ERT2* mice [34] with *Cbs*^*fl/fl*^ mice to generate *Lgr5-Cre-ERT2; Cbs*^*fl/fl*^ mice. Eight-week-old *Lgr5-Cre-ERT2; Cbs*^*fl/fl*^ and *Cbs*^*fl/fl*^ mice were treated with tamoxifen every other day for a total of three doses (Fig. 6A) to delete CBS in the colon epithelium; colorectal carcinogenesis was then induced by azoxymethane/dextran sodium sulfate (AOM/DSS) treatment [35]. Compared with control *Cbs*^*fl/fl*^ mice, *Lgr5-Cre-ERT2; Cbs*^*fl/fl*^ mice developed fewer tumors (Fig. 6B and C). In addition, tumor size was significantly reduced in *Lgr5-Cre-ERT2; Cbs*^*fl/fl*^ mice compared with *Cbs*^*fl/fl*^ mice (Fig. 6D and E). Moreover, the colorectal histological features, including colon thickening, hyperplasia, and inflammation, were ameliorated in *Lgr5-Cre-ERT2; Cbs*^*fl/fl*^ mice compared to *Cbs*^*fl/fl*^ mice (Fig. 6F). Together, these data demonstrate that ablation of CBS significantly inhibits colorectal tumorigenesis in mice.

To further elucidate the role of CBS in CRC progression, we conducted functional studies using SW480 and DLD1 cell lines with CBS KD. Analysis revealed that CBS KD significantly inhibited CRC cell proliferation (Figs. S12A and B) and colony formation (Figs. S12C and D), and that both these effects could be rescued by CBS overexpression (Figs. S12E and F). In addition, inhibition of CBS by AOAA treatment showed the same effects as CBS KD (Figs. S13A–D). Furthermore, both CBS KD (Figs. S12G-L) and AOAA treatment (Figs. S13E and F) significantly inhibited CRC cell migration and invasion. Similarly, transient knockout of CBS using CRISPR-Cas9 inhibited the proliferation and migration of CRC cells (Figs. S13G–I). Together, blocking CBS inhibits the tumorigenesis of CRC cells in vitro.

To ascertain the translation of the in vitro results to an in vivo model system, we subcutaneously injected CBS KD and control SW480 cells into nude mice and monitored the tumor growth. CBS KD significantly inhibited the growth of CRC xenografts, as indicated by the reductions in tumor volume and weight (Fig. 6G and H). Consistent with the observed differences in tumor size between groups, positive Ki-67 staining in tumors was dramatically reduced in the CBD KD group compared with the control group (Fig. 6I). Together, knockout or knockdown CBS inhibits CRC tumorigenesis.

## 3 Discussion

CBS synthesizes glutathione through the transsulfuration pathway, recent studies have shown that CBS possesses the capability to resist ferroptosis resulting from cellular oxidative stress [36,37]. In this study, we identified the transcription factor ATF3 as a positive regulator of CBS expression. Moreover, inhibition of CBS sensitizes CRC cells to ferroptosis by disrupting mitochondrial homeostasis under ferroptotic stress. Clinically, our study showed that the ATF3-CBS signaling axis is highly correlated with CRC progression. Consequently, CBS may serve as an effective target to enhance ferroptosis-based CRC cancer therapy.

In the role of CBS in regulating ferroptosis, our data indicate that there is no discernible alteration in the glutathione content in CRC cells with CBS knockdown. Although, the GSH:GSSH ratio has no change appears contradictory. It has been reported that GCLC catalyzes the reduction of cystine to cysteine along with the biosynthesis of endogenous GSH [38]. Furthermore, under conditions of cystine restriction, GCLC takes on a glutathione-independent, non-canonical function in protecting against ferroptosis through the regulation of glutamate homeostasis [39]. Moreover, de novo cysteine synthesis was highest in normal liver and absent in some tissues, whereas cysteine synthesis was either inactive or downregulated during tumorigenesis. In addition, differences in glutathione metabolic activity were evident across tumor types [40]. These disparities may arise from variations in disease models and cellular types, highlighting the complexity of the molecular mechanisms of CBS in ferroptosis. In this study, supplementation of exogenous glutathione only exhibited partial efficacy in reducing ferroptosis induced by cystine restriction. However, it failed to alleviate ferroptosis promoted by suppression of CBS. Conversely, mitochondrial homeostasis in CRC cells was detected disrupted upon knockdown of CBS contributing to tumor energy starvation and impairing growth. It has been reported that CBS modulates mitochondrial dynamics in endothelial cells mediates oxidative phosphorylation in DS cells [41,42]. In this study, the differential metabolites were enriched in the TCA cycle of whole-cell metabolites and mitochondrial metabolites in CBS knockdown CRC cells. It has been reported that alterations in mitochondrial TCA cycling play a pivotal role in promoting cystine restriction-induced ferroptosis [10]. Potentially, compensatory bypass pathways may arise in response to changes in metabolites; nevertheless, the reversal of mitochondrial damage proves challenging. Therefore, the metabolic role of CBS in ferroptosis, particularly within mitochondria, requires comprehensive studies across diverse cell types and disease models to elucidate its broader applications.

Among transcription factors regulating ferroptosis, Nrf2 was identified as capable of precisely modulate ferroptosis by targeting multiple ferroptosis cascade genes [43]. In addition, NRF2 has been reported to regulate CBS [37]. And we showed that ATF3 expression was upregulated earlier than NRF2 expression in response to ferroptosis stress. Intriguingly, depletion of ATF3 led to an earlier and more pronounced increase in ferroptosis compared to ATF4. Thus, we speculated that the transcription factor ATF3 functions as the master sensor of cystine deprivation-induced ferroptosis. Furthermore, we obtained evidence that ATF3 directly regulates CBS expression under cystine-restriction condition through CUT&Tag analysis. Similarly, we found ATF3 binding to other ferroptosis-related genes, such as ALOX15B and DUSP1. Whether these genes are also involved in cystine deprivation-induced ferroptosis should be investigated in future studies to understand global gene regulation in response to cystine deprivation comprehensively.

In recent years, with the development of technology for the metabolic characteristics of tumors in vivo, some studies have revealed that modulating specific metabolites can enhance tumor responsiveness to treatment [44]. However, the therapeutic effectiveness targeting the transsulfuration pathway, specifically cysteine metabolism, has demonstrated restricted efficacy in diverse tissues, including clinical interventions for CRC. Our data revealed that blocking CBS decelerates CRC tumorigenesis in vitro and in vivo. Simultaneously, based on blocking CBS sensitized ferroptosis in CRC cells, coupled with dietary cystine restriction, it emerges as a significant clinical target. Furthermore, ATF3 is activated in the early stages of ferroptosis, serving as an indispensable and crucial effector molecule in the entire cascade pathway. In conclusion, targeting the ATF3/CBS signaling axis along with an amino acid-restricted dietary regimen holds significant clinical potential for treating colorectal cancer.

## 4 Materials and methods

### 4.1 Patient-derived colorectal tissue samples

One cohort of CRC samples consisting of eighty-six pairs of paraffin-embedded CRC specimens and adjacent normal tissues from colon cancer patients was obtained from Shanghai Outdo Biotech Co. Ltd. Another cohort of CRC samples consisting of forty-seven pairs of paraffin-embedded surgical CRC specimens, was obtained from the First Affiliated Hospital of Xiamen University (Xiamen, China). Informed consent was obtained from all the patients. The study protocol conformed to the ethical guidelines of the Declaration of Helsinki and was approved by the Institute of Research Ethics Committee of Xiamen University.

### 4.2 Mice and housing conditions

Male BALB/c nude mice (6–8 weeks old) were used for subcutaneous tumorigenesis experiments. Male C57BL/6 mice (6–8 weeks old) were used for subcutaneous tumorigenesis experiments involving a cystine-restricted diet. *Lgr5-Cre-ERT2; Cbs*^*fl/fl*^ (B6.129P2-*Lgr5*^*tm1(cre/ERT2)Cle*^, #008875, The Jackson Laboratory) were crossed with *Cbs*^*fl/fl*^ mice (C57BL/6, NO.T009473, GemPharmatech) to ablate CBS in colonic stem cells. To induce Lgr5-Cre-ERT2-mediated recombination, 8-week-old mice were treated with 200 mg/kg tamoxifen daily by gavage for three consecutive days. Age-matched littermates were used as controls for all experiments. All the mice had a C57BL/6 background. Information on the individual mouse strains is presented in Supplementary Table 1. The Xiamen University Institutional Animal Care and Use Committee approved all the experimental procedures. Efforts have been made to minimize animal suffering.

### 4.3 Cell lines and cell culture

SW620, SW837, SW480, DLD1, and HEK293T cells were purchased from the ATCC. Wild-type and CBS KO MEFs were generated from day 13.5 embryos isolated from wild-type and CBS KO mice (C57B6, #002461, The Jackson Laboratory). DLD1 cells were cultured in RPMI-1640 medium (Shanghai Basal Media Technologies Company, L210KJ). HEK293T cells and MEFs were cultured in DMEM (Shanghai Basal Media Technologies Company, L110KJ). SW480, SW620, and SW837 cells were cultured in L-15 medium (Shanghai Basal Media Technologies Company, L620KJ). For cystine restriction experiments, cells were grown in L-cystine-free RPMI 1640 medium or in medium supplemented with 65 mg/L L-cystine. All cells were grown in a medium containing 10% fetal bovine serum (FBS; A24G00J, Gemini) supplemented with 100 units/mL penicillin and streptomycin (PSL01, Caisson). All cells were incubated at 37 °C in a humidified atmosphere containing 5% CO_2_. All cell lines were identified using short tandem repeat (STR) profiling.

### 4.4 Genotyping

Genotyping of all strains obtained from The Jackson Laboratory was performed using the protocols provided by the vendor. Genotyping of *Cbs*^*fl/fl*^ mice was performed using the following primers: forward primer, 5′-atgttgaagaggtgtcttagttggg-3’; reverse primer, 5′-caagaattaaagtgcctcagggaa-3’. The thermal cycling conditions were as follows: 94 °C for 3 min, followed by 30 cycles at 94 °C for 30 s, 57 °C for 40 s, and 72 °C for 40 s. The expected amplicons from this PCR were 435 bp for *Cbs*^*fl/fl*^ mice and 339 bp for the WT mice. The procedure for genotyping recombinant CBS mice was custom-designed with the following primers: forward primer, 5′-atgttgaagaggtgtcttagttggg-3’; reverse primer, 5′-cctgcattcatctgggacctaat-3’. The thermal cycling conditions were as follows: 94 °C for 3 min; 30 cycles at 94 °C for 30 s, 57 °C for 40 s, and 72 °C for 40 s; final extension at 72 °C for 10 min; and holding at 4 °C. The expected amplicons for this PCR were 2148 bp for unrecombined *Cbs*^*fl/fl*^ mice, 436 bp for recombined *Cbs*^*fl/fl*^ mice, and 1954 bp for WT mice. The amplicon size was determined by electrophoresis.

### 4.5 Detailed methods

#### 4.5.1 shRNA synthesis, lentiviral infection and construction of CBS knockout cell lines

shRNA sequences targeting CBS, ATF3, and ATF4 were synthesized (sequences listed in Supplemental Table 1). Oligo pairs were annealed and inserted into the pLKO.1 vector. pLKO.1, pLKO scrambled shRNA, psPAX, and pMD.2G constructs were purchased from Addgene. Plasmids containing the shCBS, shATF3, and shATF4 sequences were constructed using standard molecular cloning techniques. Lentiviruses were produced using the calcium phosphate method by cotransfection of the packaging plasmid psPAX, envelope plasmid pMD.2G, and shRNA plasmids into HEK293T cells. Viral supernatants were harvested 48–72 h after transfection and passed through a 0.45 μm filter. Target cells were infected with the viral supernatant at 40% confluence in fresh medium containing 8 mg/mL polybrene. Stably transduced SW480 and SW620 cells were selected using 4 μg/mL puromycin, and DLD1 cells were selected using 2.5 μg/mL puromycin in the culture medium. CBS sgRNA lentiviruses were generated by inserting human CBS-specific targeting sequences (Supplemental Table 1) into a lentiCRISPRv2 puro plasmid (Addgene #98290). SW480 cells were treated as described above and selected in a puromycin-containing medium to obtain stable knockout cells.

#### 4.5.2 Cell viability assay

For cell viability experiments involving cystine restriction, Erastin, or ferrostatin-1, the cells were incubated in white 96-well plates. Specifically, SW480, SW620, and SW837 cells were plated at 8 × 10^3^ cells per well; DLD1 cells were plated at 3 × 10^3^ cells per well. Cells were cultured overnight, transferred to the indicated medium, and incubated for 24 h. Cell viability was evaluated according to the protocol of the Cell Titer-Glo Kit (Promega #G9241). For viability assays, following treatment with Erastin and ferrostatin-1, cells were plated at 5 × 10^3^ cells per well in white 96-well plates, cultured overnight, and then treated with the indicated concentrations of Erastin alone or in combination with 2 μM ferrostatin-1 for 48 h. The viability assays were performed as described above.

#### 4.5.3 Cell proliferation assay

A total of 2 × 10^3^ cells per well were plated in 24-well plates and harvested daily. After collection, the cells were fixed with paraformaldehyde (PFA), stained with 0.1% crystal violet for 30 min, rinsed, and dried. After dissolving the stain by adding 30% acetic acid to the cells for 30 min, the resulting solution was transferred to a transparent 96-well plate and the absorbance was measured at 540 nm. The absorbance values measured on the first day of growth were considered the basal values, and fold changes were calculated based on the absorbance values.

#### 4.5.4 Colony formation assay

A total of 1 × 10^3^ cells were plated into each well of six-well plates with three replicates per cell type. The medium was replaced with fresh culture medium every 3 days until colonies formed. After fixing with 4% PFA for 15 min, colonies were stained with 0.1% crystal violet for 30 min, washed, and dried. The six-well plates were placed inside a scanner for image acquisition, and the colonies were counted.

#### 4.5.5 Bright field imaging of cell morphology

Cells were seeded in 6-well plates at approximately 30% confluence to observe the cell morphology during ferroptosis. After overnight culture, the medium was replaced with fresh complete or conditioned media. Cell morphology was documented using a microscope (ECHO Revolve Laboratories, RVL-100) at different time points.

#### 4.5.6 ROS measurements

##### 4.5.6.1 Lipid reactive oxygen species (lipid ROS) and mitochondria lipid ROS measurement

A total of 1.5 × 10^5^ cells were seeded in each well of a 12-well plate and cultured in fresh complete medium or conditioned medium overnight. Next, 2 μM C11 BODIPY or mitochondrial C11 BODIPY was added to the cell medium for 30 min, and the cell samples were washed twice with PBS. Cells were harvested by trypsinization and collected for flow cytometry-based analysis of lipid ROS. The cells were then resuspended in full medium (FM) or cystine-restricted (CR) medium containing 2% FBS. Fluorescence emission at 520 nm–550 nm and above 580 nm was detected using a BD Fortessa flow cytometer with 488 nm and 565 nm excitation. The signals from both non-oxidized C11 (PE channel) and oxidized C11 (FITC channel) were monitored.

For fluorescence microscopy-based analysis of lipid ROS, cell images were acquired in TRITC, FITC, and transmitted light channels using a fluorescence microscope system (ECHO Revolve Laboratories, RVL-100). Finally, the images in all the three channels were merged to generate the final images.

For the fluorescence microscopy-based analysis of mito-lipid ROS, cell images were acquired in the FITC and Deep Red channels using a fluorescence microscope system (ZEISS LSM 900 with Airyscan 2). Finally, the images in the two channels are merged to generate the final images.

##### 4.5.6.2 Mitochondrial superoxide (mitochondrial ROS level) measurements

MitoSOX (1:2000 dilution) was added to the cell medium and incubated for 30 min. The cells were washed twice with PBS, collected, and resuspended in the corresponding medium for flow cytometry analysis. Fluorescence emission at 580 nm was detected using a BD Fortessa flow cytometer with 565 nm excitation.

##### 4.5.6.3 Intracellular ROS measurements

H2DCFDA (1:1000 dilution) was added to the cell culture medium and incubated for 30 min. The cells were collected and resuspended in the corresponding medium for flow cytometry analysis. Fluorescence emission in the 505–550 nm range was detected using a BD Fortessa flow cytometer at 488 nm excitation.

#### 4.5.7 Western blot analysis

Cells were washed twice with cold PBS and then lysed with RIPA buffer (50 mM Tris (pH 8.0), 15 mM NaCl, 2 mM EDTA, 1% SDS, 0.5% sodium deoxycholate, 1% NP-40, and 1 mM PMSF) at 4 °C for 30 min. The whole-cell lysate was collected, ultrasonicated, and centrifuged at 12,000 rpm for 10 min. The soluble proteins were collected. A standard BCA kit was used to measure the protein concentration in the lysate. Proteins were separated using 10% SDS-PAGE and transferred to a polyvinylidene difluoride (PVDF) membrane. After blocking with 5% nonfat milk for 60 min, the membrane was incubated first with the indicated primary antibodies overnight at 4 °C, and then with the corresponding horseradish peroxidase-labeled secondary antibody. Protein levels were measured using a standard ECL kit and images were acquired using a C-Series Imaging System (Azure Biosystems C500).

#### 4.5.8 Cell migration and invasion assays

For the cell migration assay, 5 × 10^4^ cells were added to the top chambers of Transwell inserts with 8 μm pores (Corning) in serum-free medium. A medium containing 10% FBS was added to the bottom chambers. The cells were cultured at 37 °C for two days. Cells that remained on the upper surface of the membrane were removed using cotton swabs. The migrated cells on the bottom surface of the Transwell insert were gently washed with PBS, fixed with methanol, and stained with crystal violet for 30 min. After rising, the migrated cells on the bottom surface of the Transwell membrane were imaged. The cell invasion assay was performed similarly, except that the membranes in the upper chambers were precoated with 70 mL of diluted Matrigel Basement Membrane Matrix. Cells were randomly photographed in five fields (200 × magnification) for each experiment and data from three independent assays were used for analysis.

#### 4.5.9 Seahorse XF glycolysis assay

A total of 4 × 10^4^ cells were plated in the wells of an Agilent Seahorse 96-well XF Cell Culture Microplate. After overnight culture, the cell culture medium was replaced with an analysis medium (pH 7.4, Hippocampal Bioscience) supplemented with 0.5 mM pyruvate, 25 mM glucose, and 4 mM glutamine for oxygen consumption rate (OCR) measurement. The cells were incubated at room temperature for 30 min. Then, 1 μM oligomycin, 0.5 μM FCCP and 1 μM rotenone/antimycin were added at the specified times. For extracellular acidification rate (ECAR) measurement, the cells were cultured in analytical medium (pH 7.4) containing 4 mM glutamine (Hippocampal Bioscience) for 30 min at room temperature. Then, 12.5 mM glucose, 1 μM oligomycin, and 100 mM 2-DG were added at the specified times. The OCR and ECAR values were measured using a Seahorse XF96 instrument (Seahorse Bioscience, USA). Another sample containing the same number of cells was prepared to normalize the experimental data. The OCR and ECAR values were corrected using Seahorse XF96 Wave software.

#### 4.5.10 Transmission electron microscopy

A total of 1 × 10^6^ cells were plated in 60 mm culture dishes. After overnight culture, the medium was replaced with fresh complete medium or conditioned medium, and the cells were incubated for 12 h. Then, the cells were quickly collected at 4 °C and fixed overnight in fixation solution containing 2.5% glutaraldehyde and 0.1 mM phosphate buffer. After dehydration, the cells were embedded in an epoxy resin. Ultrathin sections were sliced using an ultramicrotome and placed on a copper mesh (300 mesh). The distribution and morphology of the mitochondria were evaluated using a transmission electron microscope (Hitachi HT-7800).

#### 4.5.11 Live-cell imaging

We generated SW480 cells labeled with the CBS-P2A-Oxstaygold [45]. Labeled SW480 cells were seeded on glass-bottom plates and treated in fresh culture media the following day. For cystine restriction conditions, cells were washed twice with PBS, and the cystine restriction medium was incubated with 5 μM SYTOX Orange prior to treatment. Imaging was performed in live-cell incubation chambers maintained at 37 °C and 5% CO_2_. Images were acquired every 10 min for 24–48 h using a Zeiss Cell discoverer 7 microscope. Time-lapse imaging and images were quantified using ZEN software (Zeiss).

#### 4.5.12 Subcutaneous xenograft model

Male nude mice aged 5–6 weeks were obtained from the Experimental Animal Center of Xiamen University. Then, 2 × 10^6^ shCtrl and shCBS SW480 cells were subcutaneously injected into the flank of the same mouse, with shCtrl cells on the left side and shCBS cells on the right side. A vernier caliper was used to measure the tumor volume along two vertical axes once every two days beginning 10 days after cell inoculation. Three weeks after inoculation, the mice were euthanized, and subcutaneous tumors were isolated for further analysis.

#### 4.5.13 Cystine restriction diet study in mice

Eight-week-old C57BL/6 mice were subcutaneously inoculated with the mouse colorectal cancer cell line MC38. As shown in the schematic diagram (Fig. 2A), the mice were fed a control diet (containing cystine) and cystine-restricted diet (without cystine) before cell inoculation for one week. Treatment was continued until the end of the experiment. Next, the two groups of mice were divided into two subgroups. When the tumor volume grew to an appropriate size, one subgroup of mice received an intraperitoneal injection of the CBS inhibitor aminooxyacetic acid (AOAA) at a dose of 9 mg/kg per day, and the other subgroup of mice was injected with the same volume of saline as a control, and the subcutaneous tumor volume of these mice was continuously monitored. When the tumors grew to an appropriate size, the mice were euthanized and the subcutaneous tumors were removed. The final volume and weight of the tumors in each mouse were measured.

#### 4.5.14 Hematoxylin and eosin (H&E) staining, immunohistochemical analysis and pathological analysis

Tissue samples from the mice were immediately rinsed with cold PBS, fixed overnight with 4% PFA, and dehydrated in increasing gradients of ethanol and xylene. The specimens were then embedded in paraffin and sliced into 5 μm sections. For H&E staining, tissue sections were dewaxed and stained with a standard hematoxylin and eosin (H&E) solution.

For immunohistochemical staining, tissue sections were dewaxed and antigen retrieval was performed in citrate buffer by heating in a boiling water bath (100 °C) in a microwave oven for 10 min. Endogenous peroxidase activity was blocked by incubating with 3% H_2_O_2_ for 10 min at room temperature. Then, Tissue sections were blocked with 5% goat serum for 30 min and then incubated with anti-CBS (1:200 dilution), anti-ATF3 (1:100 dilution), and anti-Ki67 (1:300 dilution) primary antibodies overnight at 4 °C. After rinsing, the tissue sections were incubated with biotinylated goat anti-rabbit/mouse IgG for 10 min and streptomycin-peroxidase for 10 min. DAB solution was then added for colorization. After a brief rinse, the sections were stained with hematoxylin for nuclear staining. Finally, the sections were dehydrated with ethanol and xylene and sealed with a neutral resin. Images were acquired at 100 × , 200 × , and 400 × magnifications using a Leica DM4B microscope. Samples in the negative control group were treated according to the same protocol, except that the primary antibody was replaced with the control IgG.

Immunohistochemical staining of colorectal tissue samples from patients was performed by independent pathologists. Staining intensity was scored on a scale of 0–3: negative, score = 0; weak, score = 1; moderate, score = 2; and strong, score = 3. The staining extent was scored on a scale of 1–4 based on the percentage of stained cells:0–10%, score = 1; 11–50%, score = 2; 51–80%, score = 3; and 81–100%, score = 4. The staining extent and intensity scores were multiplied to obtain the final tissue expression score for each sample.

#### 4.5.15 Immunofluorescence and confocal fluorescence microscopy

A total of 1 × 10^5^ SW480 cells or SW620 cells were seeded in 24-well plates and allowed to grow overnight before treatment. According to the requirements of the different experiments, the culture medium was replaced for a specific time period. Then, MitoTracker (1:2000 dilution) was added to the cell culture medium and the cells were incubated at 37 °C for 30 min. The cells were fixed with 4% paraformaldehyde (PFA) for 15 min at room temperature and permeabilized on ice with 0.5% Triton X-100 in PBS. The cells were blocked in PBS containing 5% BSA for 30 min at room temperature and then incubated with the appropriate primary antibody (1:200 dilution) at 4 °C overnight. Next, the cells were incubated with a FITC- or Texas Red-conjugated secondary antibody at room temperature for 1 h. Nuclei were stained with 4,6′-diamino-2-phenylindole (DAPI; Sigma–Aldrich) for 5 min. Images were acquired with a 63 × oil objective using a Leica SP8 (fluorescence) confocal microscope.

#### 4.5.16 Metabolite analysis

To determine the relative levels of intracellular metabolites, extracts were prepared and analyzed by LC/MS/MS. The control and shCBS SW480 cells were plated at a density of 3 × 10^6^ cells/dish in 100 mm tissue culture dishes overnight. Cells were trypsinized and resuspended in 1 mL of pre-cooled extraction solution (methanol/acetonitrile/water = 2/2/1) at −80 °C. The cell suspension underwent ultrasonication and freeze-thaw cycles, repeated three times. Subsequently, the lysate was transferred to a new tube after centrifugation at 14,000×*g* for 20 min. The supernatant was collected, dried using vacuum centrifugation, and the resulting samples were stored at −80 °C until further analysis. Area Ratio values obtained from the analysis were normalized by protein concentration.

#### 4.5.17 Mitochondrial metabolite analysis

This part of the experiment follows the method of Rapid immunopurification of mitochondria for metabolite profiling [46,47]. In brief, 3 × 10^6^ control and shCBS SW480 cells expressing the 3 × Myc-EGFP-OMP25 (Control-MITO) gene, and cells expressing the 3 × HA-EGFP-OMP25 (HA-MITO) gene were plated one day before the experiment, and all subsequent steps were performed at 4 °C. Cells were washed, collected in KPBS (136 mM KCl, 10 mM KH_2_PO_4_–KOH pH 7.25), and centrifuged at 1000×*g* for 2 min. After resuspending in KPBS, cells were homogenized with 15 strokes using a glass Teflon homogenizer at 1000 rpm. The homogenates were centrifuged at 1000×*g* for 3 min, and the supernatant was incubated with magnetic anti-HA beads (Thermo Fisher Scientific) on an end-over-end rotator for 3 min at 15 rpm. Beads were collected on a magnet and washed four times with KPBS. Mitochondrial metabolites were extracted by incubating the beads with extraction buffer (40:40:20 v/v/v acetonitrile/methanol/water) for 5 min. For each experiment, an input and IP fraction were taken for immunoblot analysis of mitochondrial enrichment and purity. The metabolites were analyzed as described in the whole-cell metabolite section.

#### 4.5.18 RNA sequencing (RNA-seq) analysis

The shCtrl and shCBS SW480 cells were seeded in 6-well plates at 30% confluence for overnight growth. The medium was replaced with fresh FM or CR medium. RNA extraction was performed using an RNA Extraction Reagent Kit. Three independent biological replicates were utilized for each treatment group. The RNA sequencing library was prepared according to the Illumina TruSeq protocol and sequenced on MGISEQ-2000RS or Illumina NovaSeq 6000. Bowtie was used to compare the generated RNA-seq data with the human reference genome, and the RSEM software package with default parameters was used for the analysis. Differentially expressed genes were defined by fuzzy mapping of the variance factors in the empirical Bayes hierarchical model (EBSeq) (ask BGI for a standard analysis protocol). The pathways enriched in differentially regulated genes were analyzed using gene set enrichment analysis (GSEA) (http://software.broadinstitute.org/gsea/index.jsp).

#### 4.5.19 CUT&Tag

SW480 cells were incubated in FM or CR media for 24 h. Approximately 1 × 10^5^ cells were used to construct the library. We then followed the standard instructions of the Cleavage Under Targets and Tagmentation (CUT&Tag) kit (Vazyme TD#903). In brief, cells were first incubated with concanavalin (ConA)-coated magnetic beads and then with a ChIP-grade primary antibody (anti-ATF3, 1:50 dilution) at room temperature for 2 h. Then, the cells were incubated at room temperature for 1 h with secondary antibody. pG-Tn5 transposase was then mixed with the ConA beads and incubated at room temperature for 1 h. Then, Tagmentation buffer was added and the mixture was incubated at 37 °C for 1 h. After termination of the Tagmentation reaction, DNA was extracted using ‒ phenol-chloroform-isoamyl alcohol. The PCR mix and adapters (Vazyme TD#202) were used for library amplification. The fragment library was purified with 1.2 × volume of DNA magnetic beads (Vazyme #N411). The library length distribution was checked using an Agilent 2100 Bioanalyzer and the library was sequenced on an Illumina NovaSeq 6000 instrument.

#### 4.5.20 Isolation of mitochondrial proteins

The cells were collected and resuspended in cold PBS. Next, 1 mL of mitochondrial separation reagent containing 1 mM PMSF was added to 2 × 10^7^ cells, and the mixture was incubated at 4 °C for 15 min. The cell suspension was transferred to a 1 mL glass homogenizer and disrupted approximately 50 times. The cell homogenate was centrifuged at 1000×*g* for 10 min. Next, the supernatant was transferred to another centrifuge tube and centrifuged at 3500×*g* for 10 min. The precipitate contained isolated mitochondria and the supernatant contained the cytoplasmic protein fraction without mitochondria. Next, mitochondrial proteins were purified, and the mitochondrial precipitate was resuspended in 500 μL of mitochondrial separation reagent containing 1 mM PMSF and centrifuged at 3500×*g* for 10 min. This step is repeated twice. Cytoplasmic proteins were also purified. The supernatant of the cytoplasmic protein fraction was centrifuged at 12,000×*g* for 10 min and the resulting supernatant was collected. All the centrifugation steps were carried out at 4 °C.

#### 4.5.21 Statistical analysis

All statistical analyses were performed using GraphPad Prism 8.0 (San Diego, CA, USA). Bioinformatic analysis was performed using R (version 3.61) (http://www.R-project.org/). The number of biological replicates for each experiment is indicated in the corresponding figure legend. Unless otherwise specified, all values were calculated from at least three independent biological replicates. Unless specified otherwise, all values are expressed as the mean ± Standard Error of the Mean (SEM). A two-tailed unpaired *t*-test was utilized for all analyses unless otherwise noted. For all statistical analyses between the control and experimental groups, the results are shown in the figures as follows: *p < 0.05, **p < 0.01, ***p < 0.001, ****p < 0.0001, and N.S. (not statistically significant).

## Lead contact

Further information and requests for resources and reagents should be directed to and will be fulfilled by Yongyou Zhang (yongyouzhang@xmu.edu.cn).

## Materials availability

The noncommerical materials are available upon reasonable request.

## CRediT authorship contribution statement

**Junjia Liu:** Writing – original draft, Methodology, Investigation, Formal analysis. **Xinyi Lu:** Investigation. **Siyu Zeng:** Methodology, Investigation. **Rong Fu:** Investigation. **Xindong Wang:** Methodology, Investigation. **Lingtao Luo:** Resources. **Ting Huang:** Investigation. **Xusheng Deng:** Visualization, Data curation. **Hualei Zheng:** Methodology, Investigation. **Shaoqian Ma:** Visualization, Data curation. **Dan Ning:** Methodology. **Lili Zong:** Resources. **Shu-Hai Lin:** Resources. **Yongyou Zhang:** Writing – review & editing, Supervision, Resources, Project administration, Funding acquisition, Conceptualization.

## Declaration of competing interest

The authors declare that they have no known competing financial interests or personal relationships that could have appeared to influence the work reported in this paper.

## Data Availability

Data will be made available on request.
