# Supplementary material for: ATF3-CBS signaling axis coordinates ferroptosis and tumorigenesis in colorectal cancer
Source: Redox Biol. 2024 Mar 8;71:103118. doi: 10.1016/j.redox.2024.103118 (PMC10958616; doi:10.1016/j.redox.2024.103118)

ATF3-CBS Signaling Axis Coordinates Ferroptosis and Tumorigenesis in Colorectal Cancer

Junjia Liu[1], Xinyi Lu[1], Siyu Zeng[1], Rong Fu[1], Xindong Wang[1], Lingtao Luo[4], Ting Huang[1,3], Xusheng Deng[1], Hualei Zheng[1], Shaoqian Ma[1], Dan Ning[1], Lili Zong[3], Shu-Hai Lin[1,2], Yongyou Zhang[1,2#]

[1] State Key Laboratory of Cellular Stress Biology, Innovation Center for Cell Signaling Network, Engineering Research Centre of Molecular Diagnostics of the Ministry of Education, School of Life Sciences, Xiamen University, Xiamen, Fujian 361102, China

[2] National Institute for Data Science in Health and Medicine Engineering, Faculty of Medicine and Life Sciences, Xiamen University, Xiamen, Fujian 361102, China

[3] School of Pharmaceutical Sciences, Xiamen University, Xiamen, Fujian 361102, China

[4] Department of Gastrointestinal Surgery, The First Affiliated Hospital of Xiamen University, Xiamen University, Xiamen, Fujian 361102, China

[#] Address correspondence to authors at:
School of Life Sciences, Xiamen University, Xiamen, Fujian 361102, China. Fax: 86-0592-2187363; Email: yongyouzhang@xmu.edu.cn (Dr. Yongyou Zhang)

Supplementary Figure 1. Suppression of CBS enhances sensitivity to ferroptosis in CRC cells. Related to Figure 1.

A. The protein levels of the key enzyme in the transsulfuration pathway, CBS and the system Xc^- amino acid antiporter Xct in the indicated cell lines were assayed by Western blotting. Relative protein expression was normalized to β-actin.

B and **C.** Morphology analysis of colorectal cells. The red arrows indicate ferroptotic cells (showing swollen morphology) under cystine restriction or treatment with 10μM Erastin for 12, 24 or 36h. Scale bar, 50μm.

D and **E.** Western blot analysis of CBS levels in control and CBS KD CRC cells. Relative protein expression was normalized to β-actin.

F and **G.** Morphology analysis of the control and CBS KD cells under complete and cystine-deprived medium. The red arrows indicate ferroptotic cells under cystine restriction at 24h. Scale bar, 50μm.

H. Lipid ROS production was quantified in CBS KD SW620 cells after cystine starvation for 24h or incubation in FM by C11-BODIPY staining and flow cytometric analysis.

I and **J.** Viability of SW480 and DLD1 cells treated with Erastin. The red arrows indicate ferroptotic SW480 cells after Erastin treatment. Scale bar, 50μm.

Data are presented as mean ± SEM, and $^{**}p < 0.01$, $^{***}p < 0.001$, $^{****}p < 0.0001$ compared to control group, based on two-tailed, unpaired Student's *t*-test.

Supplementary Figure 2. Knockdown of CBS or AOAA promote ferroptosis in CRC cells. Related to Figure 1.

A. Fluorescence micrographs of SW480 cells under cystine starvation alone or in combination with 2μM Fer-1 treatment for 24h. The blue box indicates the oxidized form. Scale bar, 100μm.

B and **C.** Lipid ROS production was quantified in SW620 cells after cystine restriction alone or in combination with 2μM Fer-1 treatment for 24h by C11-BODIPY staining and flow cytometric analysis.

D and **E.** Lipid ROS production was quantified in DLD1 cells after treatment with 0, 100, 200 or 500μM AOAA alone or in combination with cystine restriction for 24h by C11-BODIPY staining and flow cytometric analysis.

F. Micrographs of DLD1 cells after treatment with 0, 100, 200 or 500μM AOAA alone or in combination with cystine restriction for 24h. The red arrows indicate ferroptotic cells. Scale bar, 50μm.

G. Flow cytometry of C11-BODIPY fluorescence in SW480 cells after treatment with conditions from panel Fig.1H.

H and **I.** Western blot analysis of CBS levels and cell viability of MEFs in the control group and CBS overexpression group under cystine restriction conditions.

J and **K.** Micrographs and cell viability of MEFs in the control group and CBS overexpression group after Erastin treatment. Scale bar, 50μm.

Data are presented as mean ± SEM, and $***p < 0.001$, $****p < 0.0001$ compared to the indicated two groups, based on two-tailed, unpaired Student's *t*-test.

Supplementary Figure 3. Supplementation of cysteine or GSH can only partially mitigate ferroptosis induced by CBS KD. Related to Figure 3.

A. Schematic of cellular strategies for cysteine acquisition, including the transsulfuration pathway and the system Xc^- amino acid transporter.

B. KEGG pathway enrichment analysis of differentially accumulated metabolites. The y-axis shows the pathway names; the x-axis shows the enrichment factor for each pathway. The bubble size indicates the number of metabolites. The color bar indicates the corrected *p* values; blue indicates a higher value, and red indicates a lower value.

C. LC-MS data was subjected to principal component analysis (PCA).

D. GSH/GSSG ratios were determined in the control and CBS KD SW480 cells.

E. Viability of CBS KD SW480 cells in response to supplementation with cysteine at different concentrations for 24h. The red arrows indicate ferroptotic cells.

F-I. Morphology, cell viability and lipid ROS levels were quantified in CBS KD SW480 cells treated with different concentrations of GSH for 24h. Scale bar, 50 μm.

J-L. Cell viability and lipid ROS production were quantified in CBS KD SW620 cells treated with GSH at different concentrations for 24h.

Data are presented as mean ± SEM, and *$p < 0.05$, **$p < 0.01$, ***$p < 0.001$, ****$p < 0.0001$ compared to control group, based on two-tailed, unpaired Student's *t*-test.

Supplementary Figure 4. Inhibiting CBS disrupts mitochondrial homeostasis. Related to Figure 3.

A. Mitochondrial stress in the indicated cells in Fig. 3B was quantified (thirty cells per group).

B and **C.** ROS in control and CBS KD SW480 and SW620 cells were quantified by H2DCFDA staining and flow cytometric analysis. The cells were treated with FM or CR medium for 24h before analysis.

D. Immunoblot analysis of whole-cell lysates (whole-cell) and lysates of mitochondria purified with anti-HA beads (anti-HA IP). Lysates were derived from SW480 cells expressing 3×Myc-EGFP-OMP25 (Control-MITO cells) or 3×HA-EGFP-OMP25 (HA-MITO cells) by rapid immunopurification of mitochondria. The corresponding subcellular compartments appear to the left.

E. KEGG pathway enrichment analysis of differentially accumulated mitochondrial metabolites.

Data are presented as mean ± SEM, and **$p < 0.01$, ***$p < 0.001$ compared to the indicated two groups, based on two-tailed, unpaired Student's *t*-test.

Supplementary Figure 5. Knockdown of ATF3 enhances sensitivity to ferroptosis in CRC cells. Related to Figure 4.

A. Western blot analysis of CBS and ATF3 in ATF3 KD SW620 cells with or without cystine restriction for 24h. Relative protein expression was normalized to β-actin.

B and **C.** Curves showing the growth of SW480 and SW620 cells in the control group and ATF3 KD group. The values for each group shown on the curves were fold-change normalized to those measured on the first day.

D and **E.** Micrographs of control and ATF3 KD SW480 and SW620 cells under cystine restriction for 12, 24 or 36h. Red arrows indicate ferroptotic cells. Scale bar, 50μm.

F. Viability of control and ATF3 KD SW620 cells under cystine restriction for 24h or incubated in FM.

G. Lipid ROS was quantified in ATF3 KD SW480 cells after cystine restriction treatment for 24h by C11-BODIPY staining followed by flow cytometric analysis.

H and **I.** Lipid ROS was quantified in ATF3 KD SW480 cells after Erastin or Sorafenib treatment for 24h by C11-BODIPY staining followed by flow cytometric analysis.

J. Micrographs of SW480 cells with ATF3 knockdown or ATF4 knockdown under cystine restriction for 24h. The red arrows indicate ferroptotic cells under CR conditions. Scale bar, 50μm.

K. Lipid ROS production was quantified in ATF3 KD and ATF4 KD SW480 cells after cystine starvation or cultured in FM for 24h alone or in combination with Fer-1 treatment as indicated by C11-BODIPY staining and flow cytometric analysis.

L. Lipid ROS production was quantified in ATF3 KD and ATF4 KD SW480 cells after cystine starvation for different time points by C11-BODIPY staining and flow cytometric analysis.

Data are presented as mean ± SEM, and *$p < 0.05$, **$p < 0.01$, ***$p < 0.001$, ****$p < 0.0001$; "ns" indicates not significant compared to control group or the indicated two groups, based on two-tailed, unpaired Student's *t*-test.

Supplementary Figure 6. CBS overexpression partially rescued ferroptosis induced by ATF3 KD. Related to Figure 4.

A and **B.** Colony formation analysis of control and ATF3 KD SW480 cells with or without CBS overexpression.

C. Micrographs of control and ATF3 KD SW480 cells with CBS overexpression under cystine restriction for 12h. Ferroptotic cells are indicated with red arrows. Scale bar, 50μm.

D and **E.** Viability of control and ATF3 KD SW480 cells overexpressing CBS and treated with Erastin or Sorafenib for 24h.

F. Lipid ROS production was quantified in ATF3 KD SW620 cells overexpressing CBS after cystine restriction for 24h by C11-BODIPY staining and flow cytometric analysis.

G-J. Lipid ROS production was quantified in ATF3 KD SW480 cells or SW620 cells overexpressing CBS after Sorafenib or Erastin treatment for 24h by C11-BODIPY staining and flow cytometric analysis.

Data are presented as mean ± SEM, and *$p < 0.05$, **$p < 0.01$, ***$p < 0.001$, ****$p < 0.0001$ compared to control group or the indicated two groups, based on two-tailed, unpaired Student's *t*-test.

Supplementary Figure 7. CBS is highly expressed in human colon adenocarcinoma in tissue microarray. Related to Figure 5.

A. IHC staining of CBS in cohort #1 microarray of colon cancer patient tissues (n=86). Representative staining of CBS in adjacent normal tissues and colon cancer foci.

B. Staining score of CBS expression in cohort #1 microarray of colon cancer patient tissues (n=86). The scores are based on the intensity and extent (area) of staining (protein expression). The immunoreactive score (ranging from 0-12) was calculated by multiplying the positive cell proportion score (1-4) by the staining intensity score (0-3).

Supplementary Figure 8. ATF3 is highly expressed in human colon adenocarcinoma in tissue microarray. Related to Figure 5.

A. IHC staining of ATF3 in the cohort #1 microarray of colon cancer patient tissues (n=86). Representative staining of ATF3 in adjacent normal tissues and colon cancer foci.

B. Staining score of ATF3 expression in cohort #1 microarray of colon cancer patient tissues (n=86). The scores are based on the intensity and extent (area) of staining (protein expression). The immunoreactive score (ranging from 0-16) was calculated by multiplying the positive cell proportion score (1-4) by the staining intensity score (0-4).

Supplementary Figure 9. CBS is highly expressed in human colon adenocarcinoma in CRC specimens. Related to Figure 5.

IHC staining of CBS in cohort #2 colon cancer patient tissues. Representative staining of CBS expression in adjacent normal tissues and colon cancer foci. The scores are based on the intensity and extent (area) of staining (protein expression). Scale bar, 50μm.

Supplementary Figure 10. ATF3 is highly expressed in human colon adenocarcinoma in CRC specimens. Related to Figure 5.

IHC staining of ATF3 in cohort #2 colon cancer patient tissues. Representative staining of ATF3 expression in adjacent normal tissues and colon cancer foci. The scores are based on the intensity and extent (area) of staining (protein expression). Scale bar, 50μm.

Supplementary Figure 11. Elevated CBS expression correlates with poorer patient survival. Related to Figure 5.

A. Kaplan-Meier analysis of overall survival in a set of CRC patients according to CBS expression using Log rank test.

Supplementary Figure 12. Suppression of CBS inhibits cell proliferation, migration, and invasion in CRC cells. Related to Figure 6.

A and **B.** Growth assay in control and CBS KD DLD1 and SW480 cells. The values for each group shown on the curves were normalized to the number of cells on the first day.

C and **D.** Representative images showing cell colony formation and the quantification of cell colonies in control and CBS KD groups of DLD1 and SW480 cells. The initial number of cells plated in each well was eight hundred.

E. Western blot analysis of CBS levels and curves showing the fold change in the population of SW480 CBS KD cells in which CBS overexpression was rescued. The values for each group shown on the curves were normalized to those measured on the first day.

F. Representative images and the quantification of colonies formed by the indicated SW480 cells in which CBS overexpression was rescued. The initial number of cells plated in each well was one thousand.

G and **H.** Scratch wound assay of control and CBS KD cells. The gray line indicates the scratch-wound boundary.

I-L. The migration and invasion potential of CBS KD and control SW480 and DLD1 cells were analyzed via Transwell assays. The results were documented and quantified as indicated. Scale bar, 50μm.

Data are presented as mean ± SEM, and $*p < 0.05$, $**p < 0.01$, $***p < 0.001$, $****p < 0.0001$ compared to control group, based on two-tailed, unpaired Student's *t*-test.

Supplementary Figure 13. AOAA inhibits cell proliferation and migration in CRC cells. Related to Figure 6.

A and **C.** Growth assay of SW480 and DLD1 cells after control (PBS) or AOAA treatment. The values for each group shown on the curves were normalized to the cell number on the first day.

B and **D.** Cell colony formation analysis of SW480 and DLD1 cells treated with control (PBS) or AOAA. The initial number of cells plated in each well was eight hundred.

E and **F.** The migration ability of the indicated cells was analyzed via a scratch wound assay. The results were documented and quantified as indicated.

G. Western blot of CBS expression in control or pooled CBS knockout SW480 cells.

H. Representative images of cell colonies and the quantification of colonies formed by SW480 CBS knockout cells. The initial number of cells in each group was one thousand.

I. Representative images of the Transwell migration and invasion assays with CBS knockout SW480 cells. Scale bar, 50μm.

Data are presented as mean ± SEM, and $*p < 0.05$, $**p < 0.01$, $***p < 0.001$, $****p < 0.0001$ compared to PBS group or control group, based on two-tailed, unpaired Student's *t*-test.

Supplemental Fig. 1

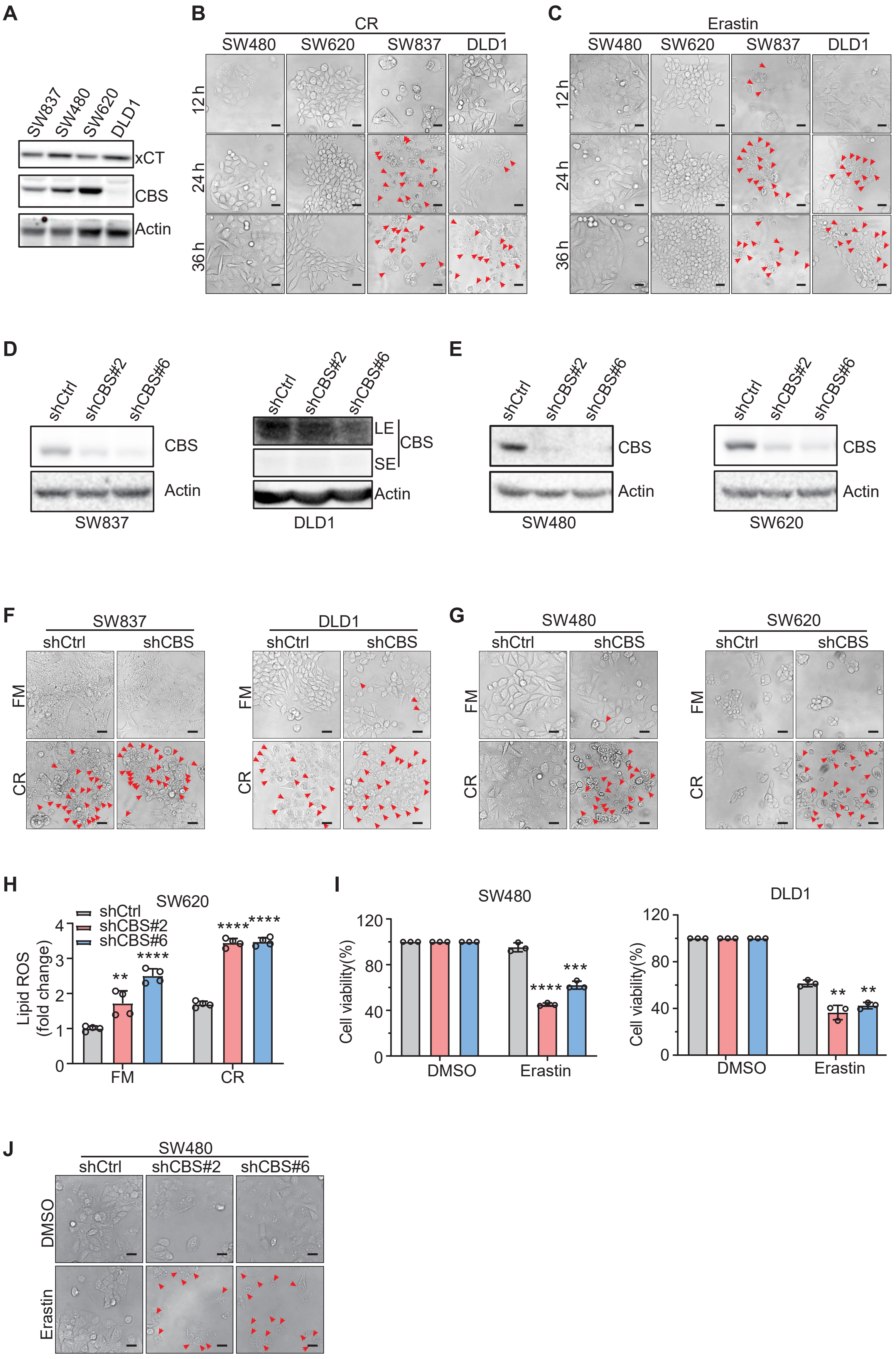

Supplemental Fig. 2

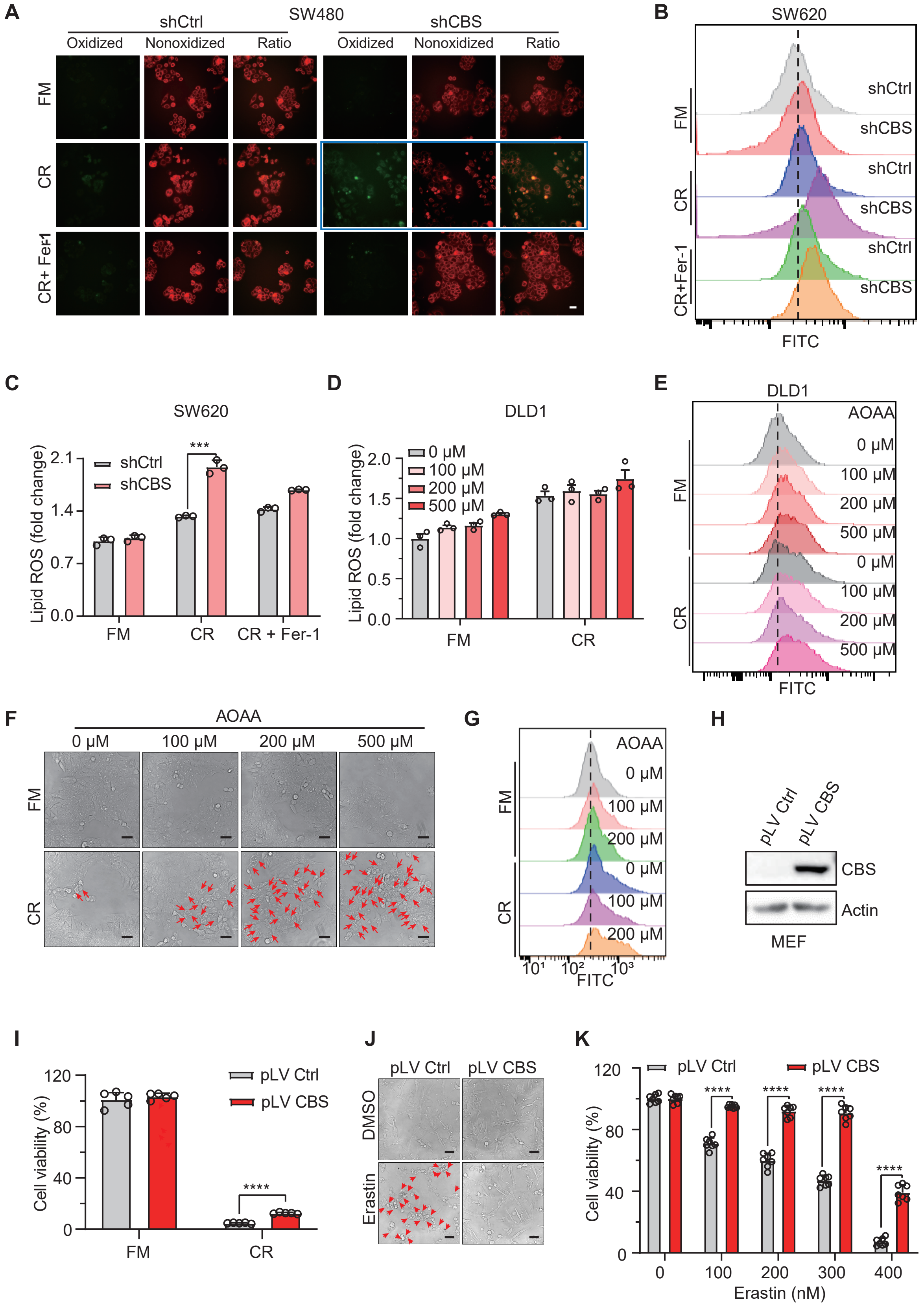

Supplemental Fig. 3

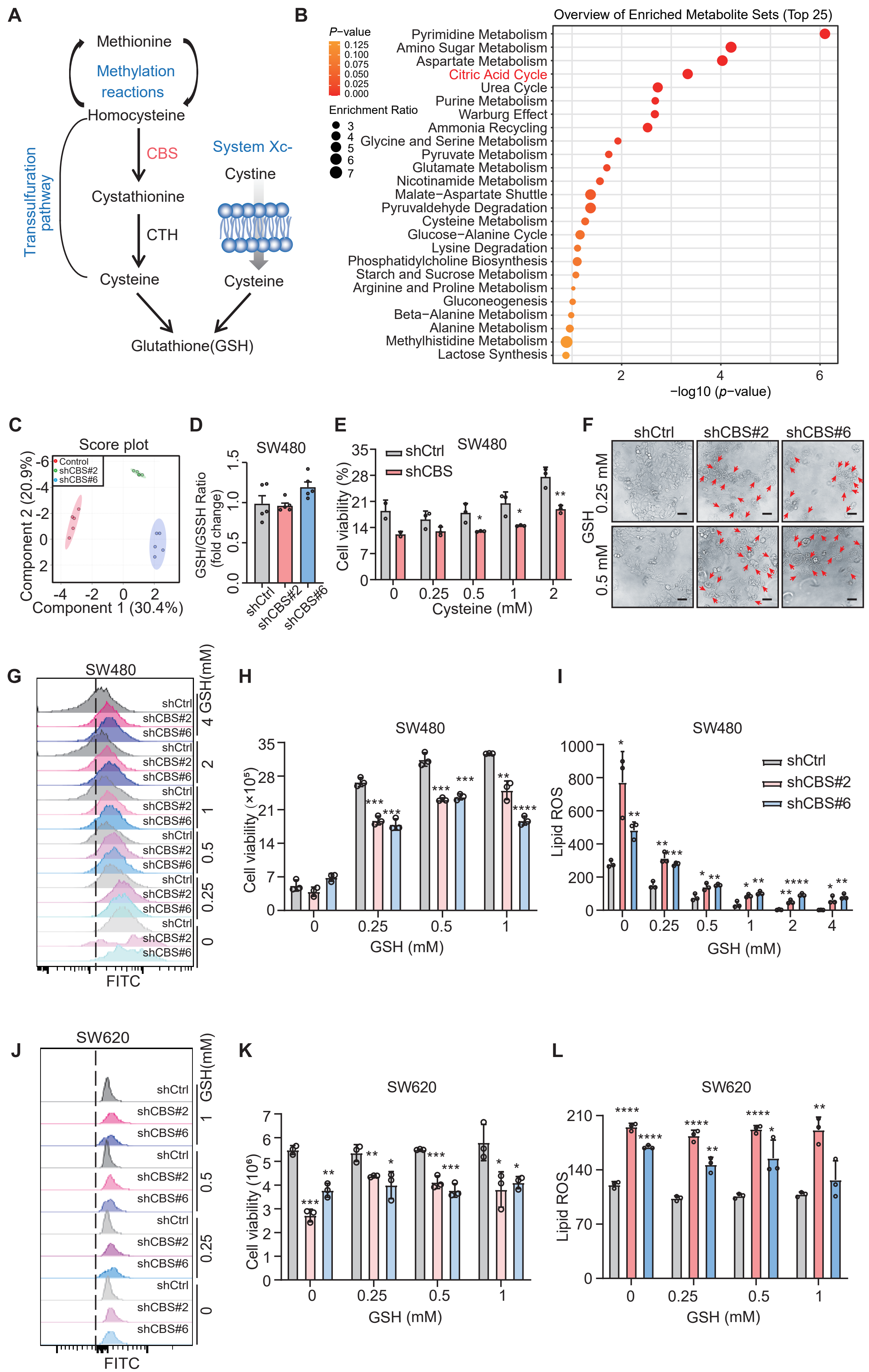

Supplemental Fig. 4

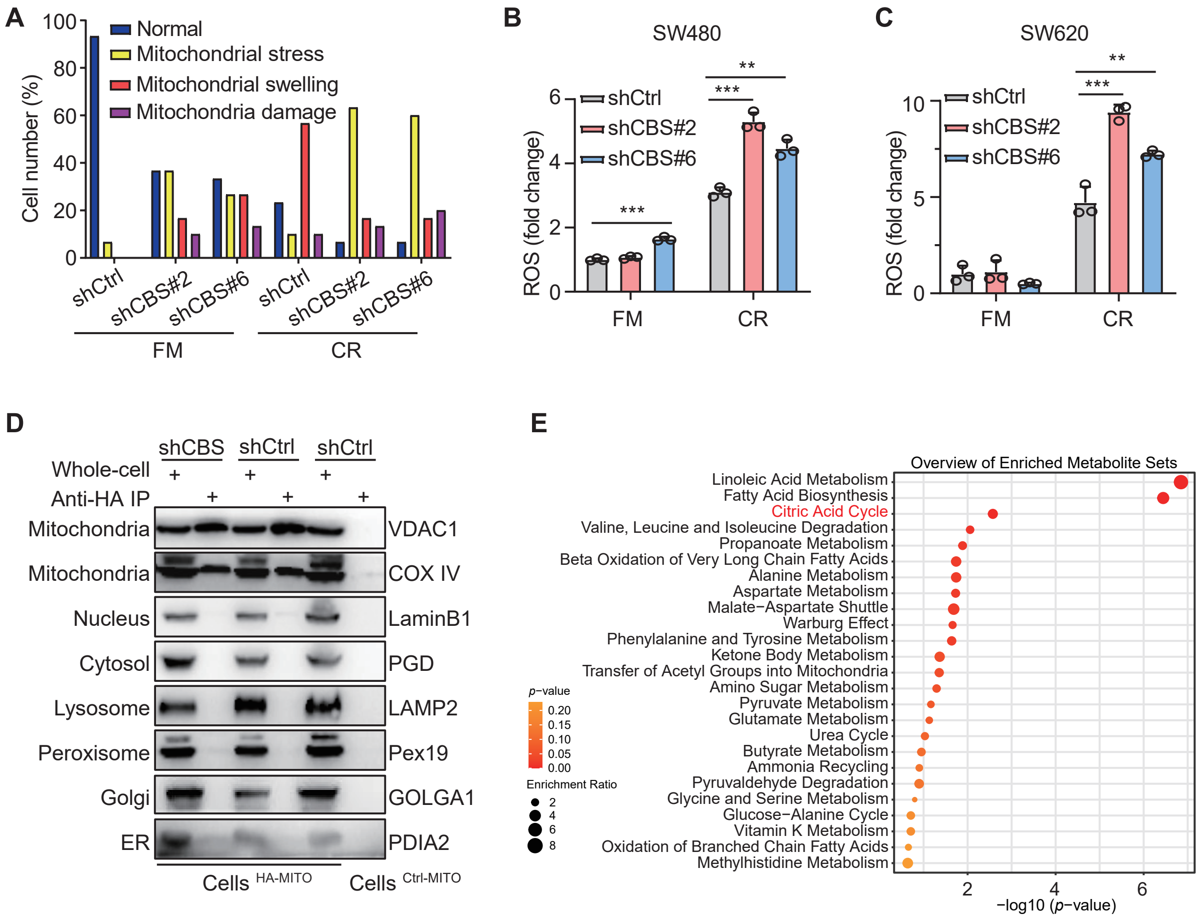

Supplemental Fig. 5

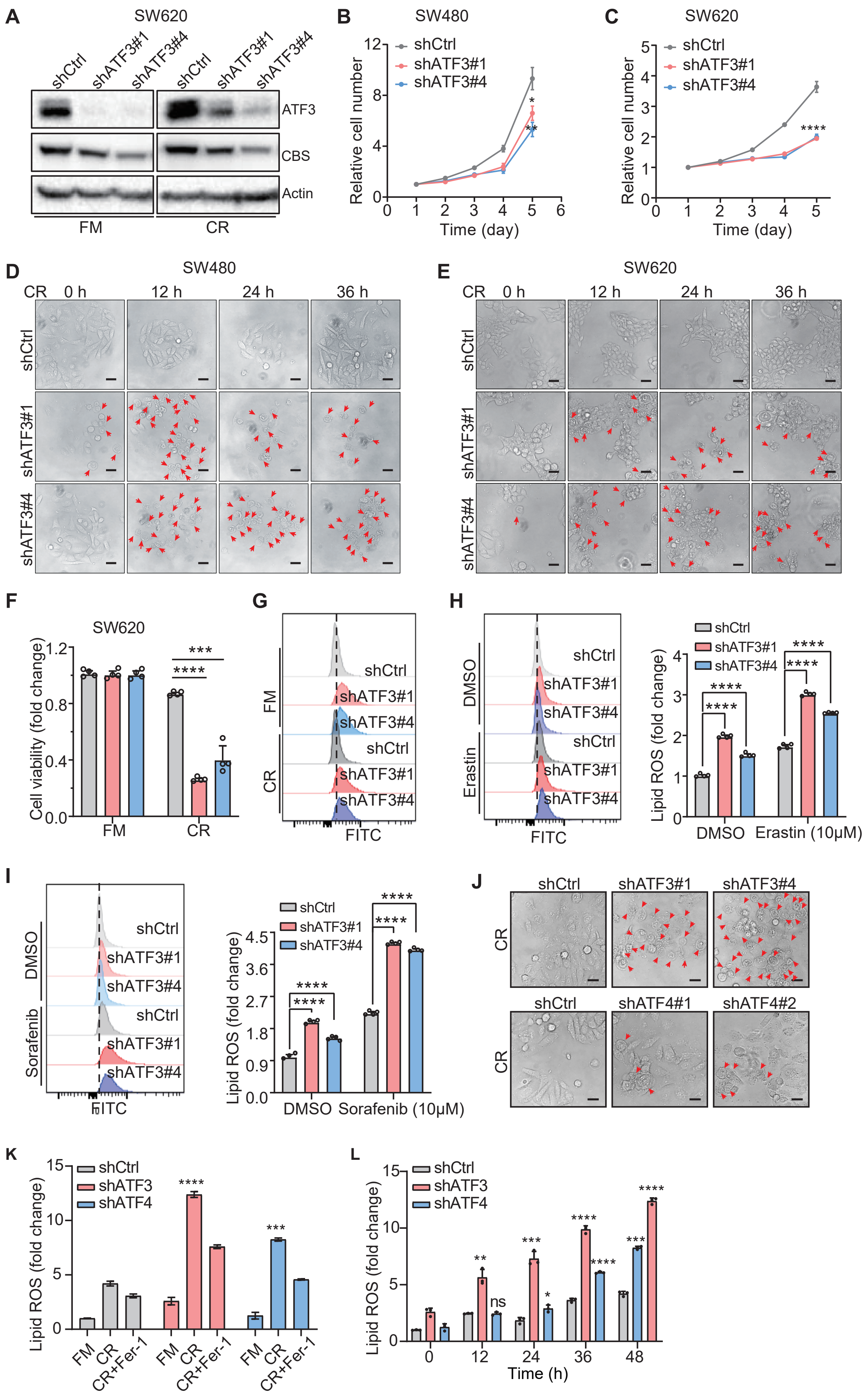

Supplemental Fig. 6

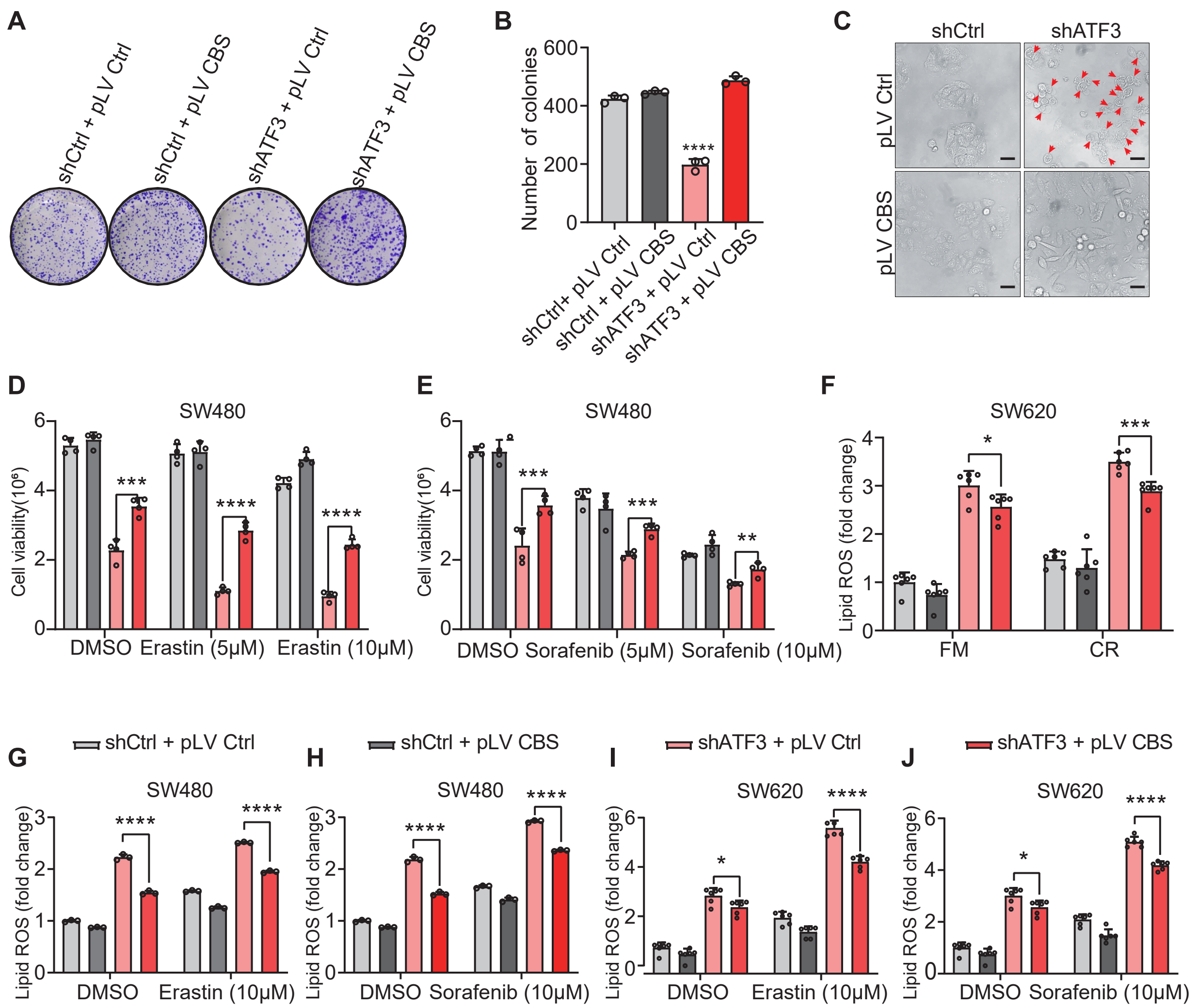

Supplemental Fig. 7

A

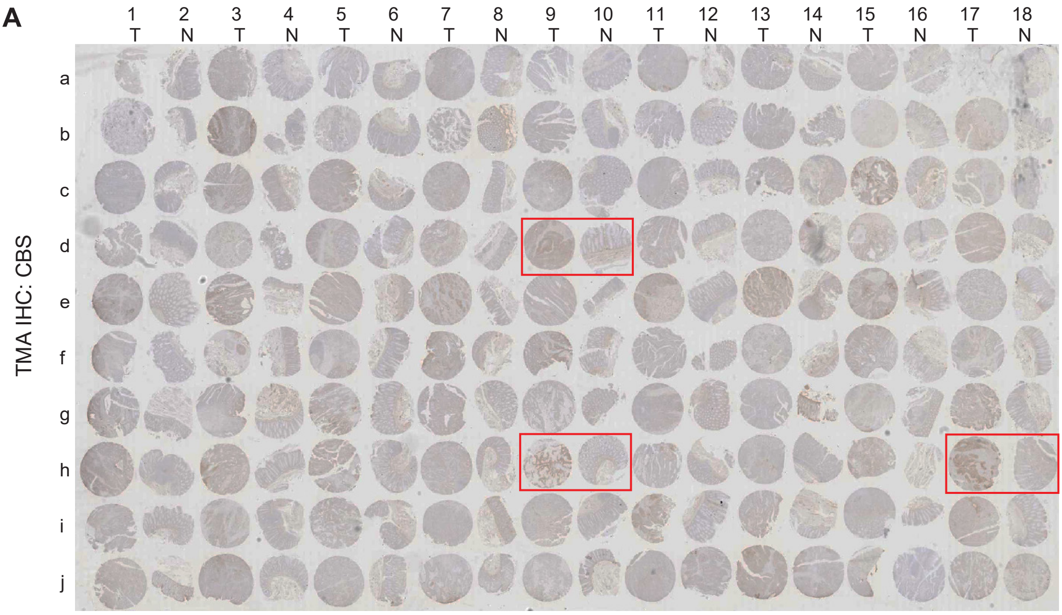

B

CBS IHC Score

	1	2	3	4	5	6	7	8	9	10	11	12	13	14	15	16	17	18
	T	N	T	N	T	N	T	N	T	N	T	N	T	N	T	N	T	N
a	4	1	6	1	0	1	6	0	0	0	4	0	3	1	3	0	3	0
b	1	1	12	1	1	1	9	3	6	0	1	0	6	2	4	2	9	1
c	2	1	12	6	8	2	8	1	12	2	4	1	8	4	12	1	8	1
d	3	1	4	2	9	1	8	2	12	3	8	1	4	3	8	2	8	1
e	8	4	12	2	8	6	12	2	8	1	12	2	12	3	12	2	6	1
f	6	1	9	4	8	1	12	2	12	2	8	1	6	3	12	2	8	1
g	6	6	4	1	12	1	12	6	8	2	8	2	4	3	8	2	8	1
h	8	1	12	1	12	2	8	4	12	2	6	3	3	2	8	0	12	1
i	4	2	9	4	8	2	8	4	8	2	12	3	8	1	6	0	8	1
j	8	3	9	1	4	2	6	4	3	2	8	8	12	6	8	0	8	8

Supplemental Fig. 8

A

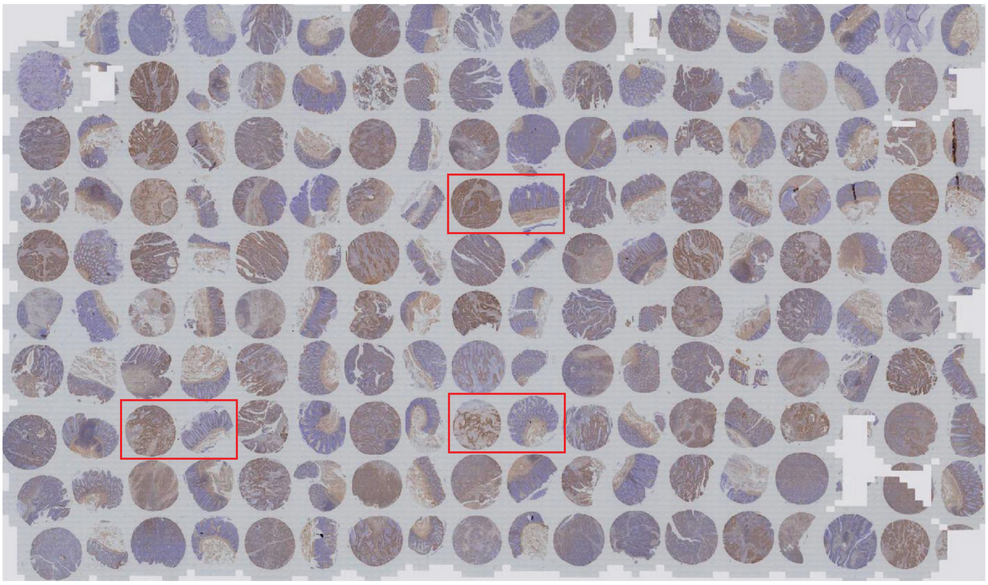

B

ATF3 IHC Score

	1	2	3	4	5	6	7	8	9	10	11	12	13	14	15	16	17	18
	T	N	T	N	T	N	T	N	T	N	T	N	T	N	T	N	T	N
a	X	1	3	2	6	2	12	4	2	0	4	0	2	6	12	2	8	2
b	1	X	12	3	8	3	16	12	6	1	9	2	9	1	4	1	6	1
c	6	1	12	9	6	1	9	0	16	4	12	4	12	4	9	2	12	3
d	4	1	16	4	12	3	9	2	16	3	4	4	2	1	6	1	16	2
e	6	4	16	2	12	4	16	3	6	1	9	12	16	9	12	3	9	1
f	1	0	9	3	4	1	12	12	16	3	9	4	12	8	4	3	3	X
g	3	8	6	3	9	6	6	4	9	1	4	12	8	16	8	9	16	9
h	3	2	16	4	6	3	12	2	16	4	12	4	8	12	16	X	6	0
i	1	0	12	2	16	8	12	0	8	4	16	3	12	3	4	X	9	6
j	3	1	12	4	8	3	8	2	12	1	4	4	2	1	6	4	12	X

Supplemental Fig. 9

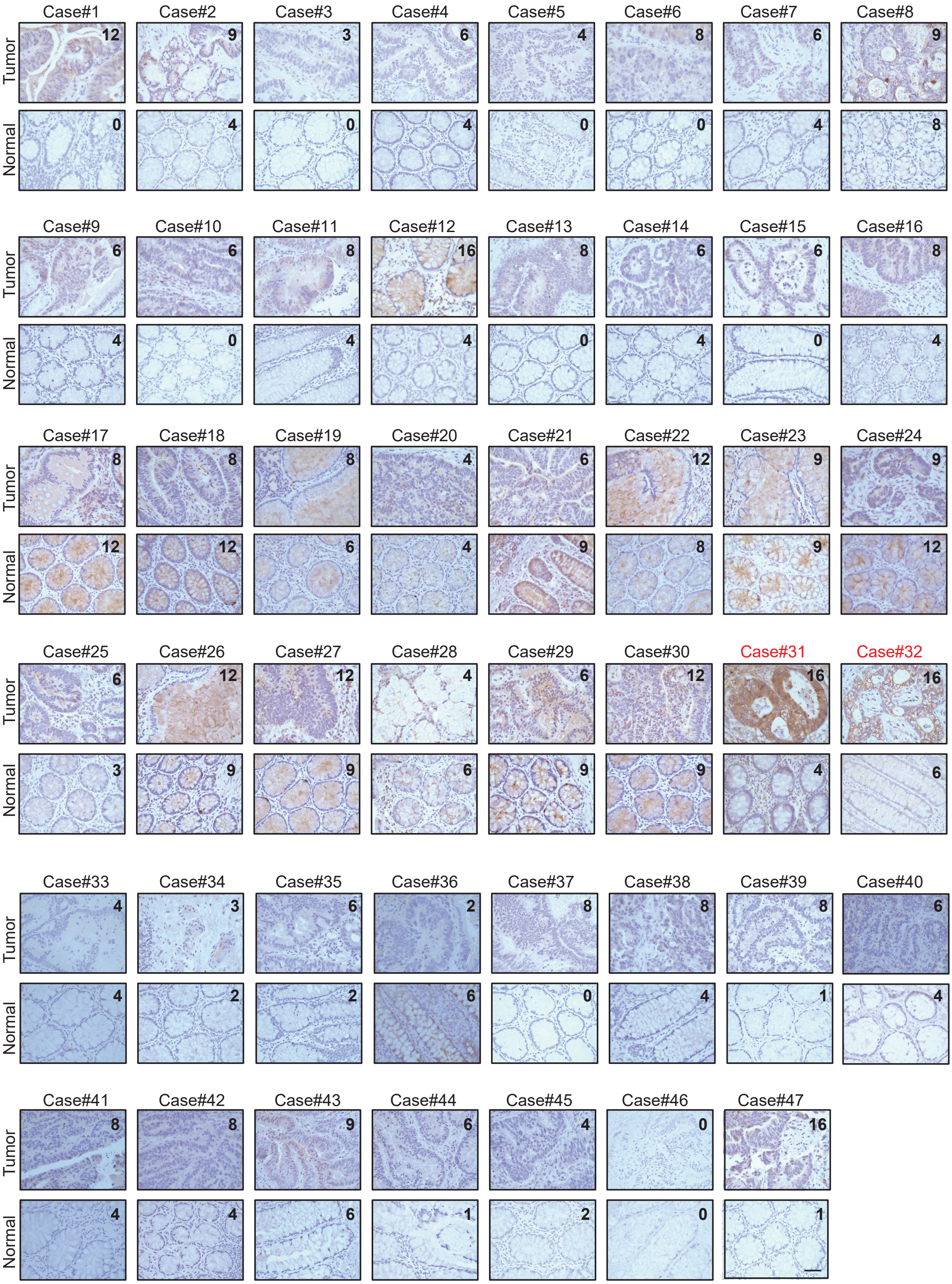

Supplemental Fig. 10

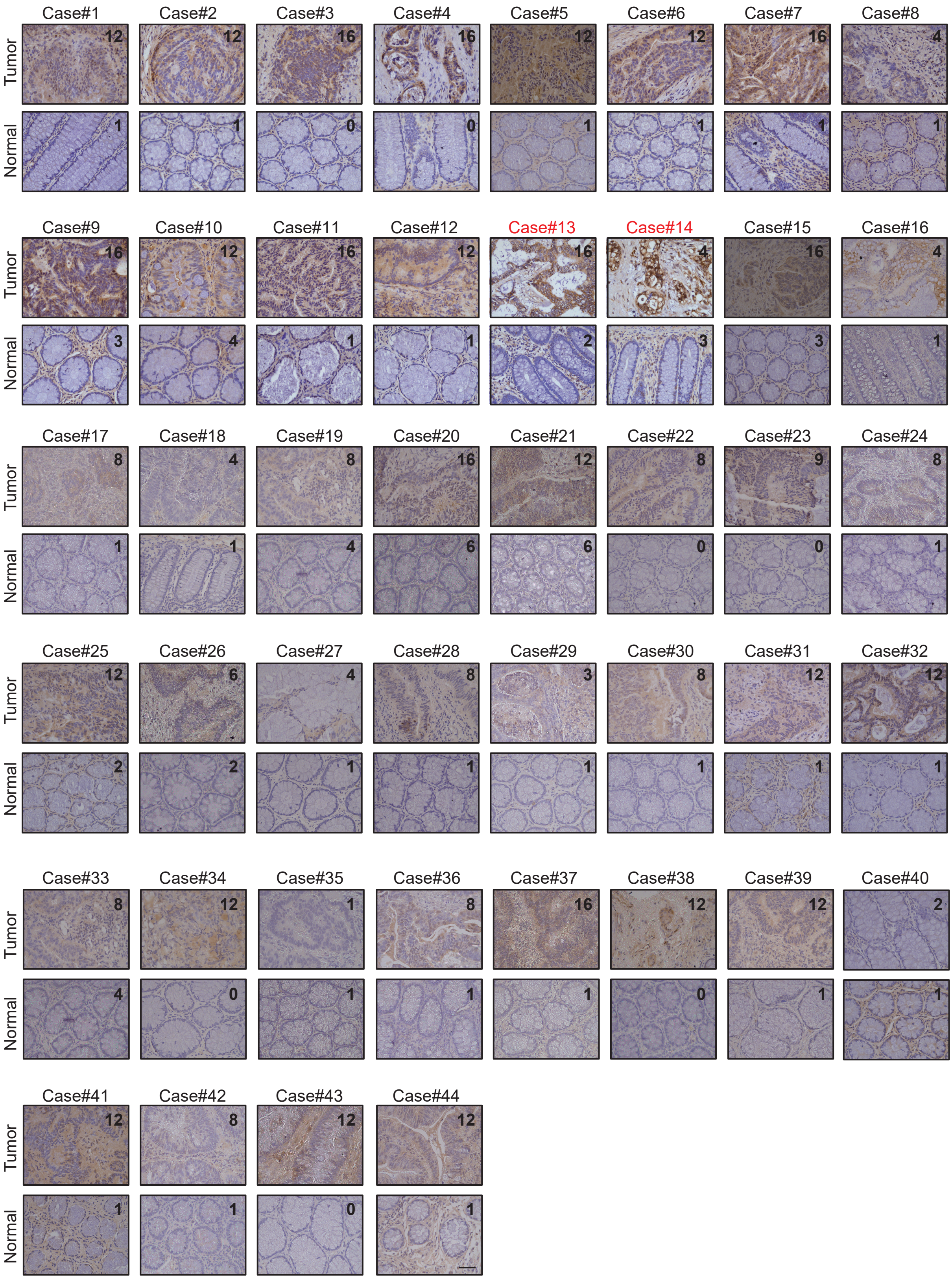

Supplemental Fig. 11

A

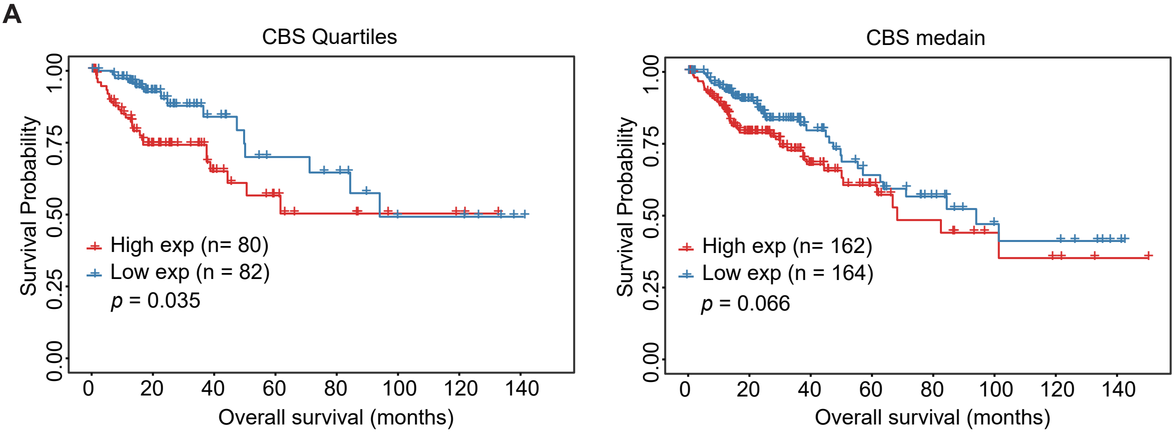

Supplemental Fig. 12

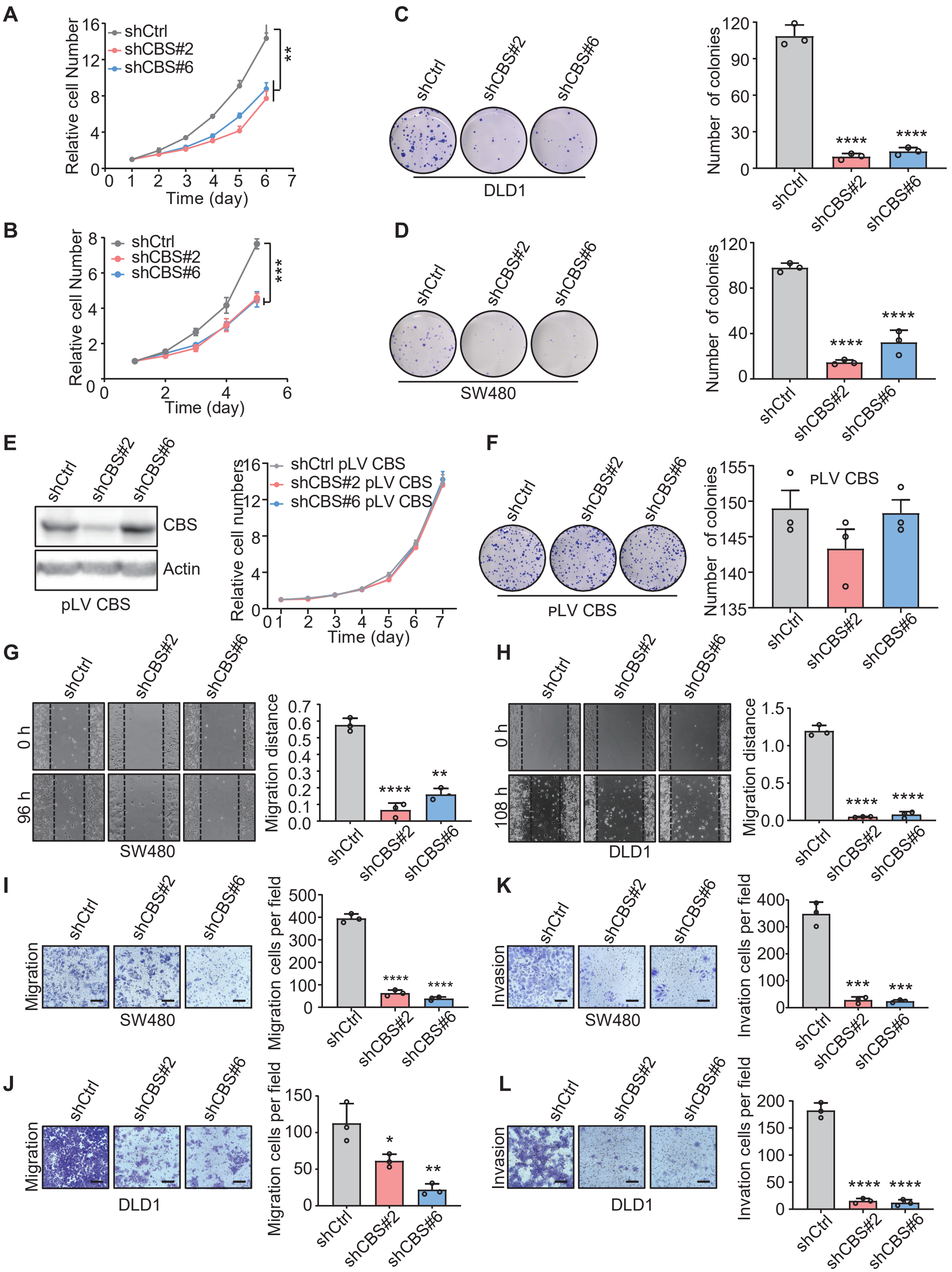

Supplemental Fig. 13

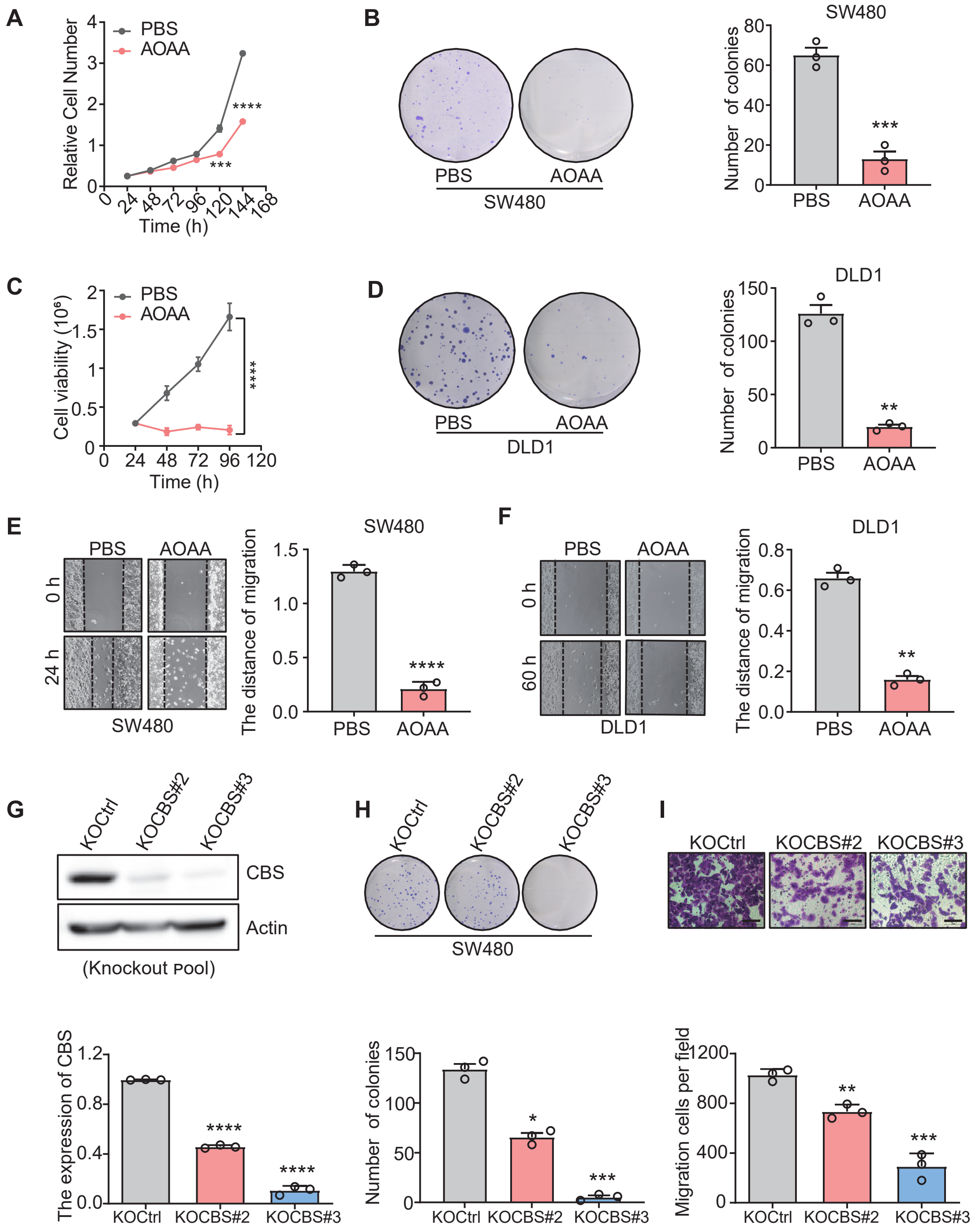

Supplement: Multimedia component 1 [file mmc1.pdf]
