# Supplementary material for: ATF3-CBS signaling axis coordinates ferroptosis and tumorigenesis in colorectal cancer
Source: Redox Biol. 2024 Mar 8;71:103118. doi: 10.1016/j.redox.2024.103118 (PMC10958616; doi:10.1016/j.redox.2024.103118)
Supplement: Multimedia component 2 [file mmc2.docx]

##

#### Supplementary Table 1: Key Resources Table

| **REAGENT or RESOURCE** | **SOURCE** | **IDENTIFIER** |
| --- | --- | --- |
| **Antibodies** |  |  |
| β-Actin (mouse) | Sigma | A5441 |
| CBS (rabbit) | Cell Signaling Technology, Abcam, Proteintech | 14782/ab231778/14787-1-AP |
| CTH (rabbit) | Proteintech | 12217-1-AP |
| ATF3 (rabbit) ChIP Grade | Abcam | ab254268 |
| ATF3 (rabbit) | ABclonal | A13469 |
| Nrf2 (rabbit) | Abcam | ab62352 |
| ATF4 (rabbit) | Proteintech | 10835-1-AP |
| GCN2 (rabbit) | ABclonal | A2307 |
| Xct (rabbit) | Abcam | Ab175186 |
| GPX4 (rabbit) | Proteintech | 14432-1-AP |
| LAMP2 (rabbit) | ABclonal | A0593 |
| PEX19 (rabbit) | ABclonal | A19237 |
| PGD (rabbit) | ABclonal | A0563 |
| Lamin B1 (rabbit) | ABclonal | A11495 |
| VDAC1 (rabbit) | ABclonal | A19707 |
| GOLGA1 (rabbit) | ABclonal | A14688 |
| PDIA2 (rabbit) | ABclonal | A12789 |
| TOMM20 (rabbit) | Cell Signaling Technology | Cat# 42406 S |
| 4-HNE (rabbit) | Abcam | Ab46545 |
| β-tubulin (mouse) | SAB | # 38075 |
| Ki-67 (human) | DakoCytomation | 00006883 |
| Goat anti-rabbit secondary antibody | Thermo Fisher Scientific | # 31210 |
| Goat anti-mouse secondary antibody | Thermo Fisher Scientific | # 31160 |
| **Clinical Samples** |  |  |
| Human colon cancer tissue samples | First Affiliated Hospital of Xiamen University | N/A |
| Human CRC tissue microarray | Shanghai Outdo Biotech Co. Ltd. | N/A |
| **Cell Lines** |  |  |
| HEK293T, SW837, SW620 | ATCC | N/A |
| SW480 | Shanghai Institute of Biochemistry and Cell Biology | N/A |
| DLD1 | Guangzhou Cellcook Biotech Company | N/A |
|  |  |  |
| **Chemicals and Culture Media** |  |  |
| L-Cystine | Sangon BioTech | A610088-0100 |
| L-Glutamine | BBI Life Science | GB0224 |
| L-Methionine | Sangon BioTech | A610346-0100 |
| Reduced GSH | Sangon BioTech | GB0229 |
| RPMI 1640 | Shanghai Basal Media Technologies Company | L210KJ |
| RPMI 1640 SAA-free | Shanghai Basal Media Technologies Company | X07881 |
| DMEM | Shanghai Basal Media Technologies Company | L110KJ |
| L-15 | Shanghai Basal Media Technologies Company | L620KJ |
| Fetal bovine serum (FBS) | GEMINI | A24G00J |
| Trypsin | BBI | A620627-0250 |
| HEPES | Sangon BioTech | A600264-0250 |
| Puromycin | Solarbio | P8230 |
| TritonX-100 | Solarbio | T8200 |
| AOAA | ALORICH Chemistry | C13408 |
| AOM | Sigma | A5486 |
| Tamoxifen | MCE | HY-13757A |
| Dimethyl sulfoxide (DMSO) | MP-Bio | 0219605580 |
| Erastin | GLPBio | GC16630 |
| Ferrostatin-1 | MackLin | F864515 |
| RNA Simple Total RNA Kit | Tiangen Biotech | DP419 |
| Sodium dodecyl sulfate (SDS) | Biosharp | BS088 |
| Isopropyl-β-D-thiogalactoside (IPTG) | BBI Life Sciences | A600168-0005 |
| Polyvinylidene fluoride (PVDF) | Merck Millipore | IPVH 00010 |
| Protease inhibitor cocktail | Roche | 04693132001 |
| Clarity Western ECL substrate | Bio-Rad | 1705060 |
| Matrigel | Corning | 356234 |
| Cell Titer-Glo | Promega | G7573 |
| SYTOX Orange | Molecular Probes | S11368 |
| C11 BODIPY | Cayman Chemical | 217075-36-0 |
| MitoPerOx | Cayman Chemical | 18798 |
| MitoSOX | Yeasen | 40778ES50 |
| MitoTracker Red CM-H2XRos | Yeasen | 40740ES50 |
| H2DCFDA | Beyotime | S0033S |
| BCA Protein Quantification Kit | Tiangen Biotech | PA115-02 |
| Cell Mitochondrial Isolation Kit | Beyotime | C3601 |
| EndoFree Mini Plasmid Kit | Tiangen Biotech | DP118-02 |
| FastQuant RT Kit | Tiangen Biotech | Cat# KR106 |
| Hyperactive In-Situ ChIP Library Prep Kit for Illumina (pG-Tn5) | Vazyme | TD901-01 |
| Costar Transwells | Corning | 3422 |
| Ultrasensitive TM SP Kit (mouse/rabbit) | MXB Biotechnologies | KIT-9720 |
| DAB Kit | MXB Biotechnologies | DAB-0031 |
| Adapter | Vazyme | TD # 202 |
| VAHTS® DNA Clean Beads | Vazyme | #N411 |
| CUT&Tag Kit | Vazyme | TD903 |
| **Oligos** |  |  |
| h. shRNA targeting sequence CBS#2: GCGGAACTACATGACCAAGTT | This paper | N/A |
| h. shRNA targeting sequence CBS #6: GACTGCGCAGAGTGGATTAAA | This paper | N/A |
| h. shRNA targeting sequence ATF3 #1: GGCTGTTGTCATACTTCTCATGG | This paper | N/A |
| h. shRNA targeting sequence ATF3 #4: GGCTGTTGTCATACTTCTCATGG | This paper | N/A |
| **Recombinant DNA (plasmids)** |  |  |
| PLV CBS binding site | This paper | N/A |
| LentiV2-human KOCBS#2 | This paper | N/A |
| LentiV2-human KOCBS#3 | This paper | N/A |
|  |  |  |
| **Software and Algorithms** |  |  |
| Prism 8 | GraphPad |  |
| ImageJ | <https://imagej.nih.gov/ij> |  |
| R3.6.1 |  |  |
| R package: ClusterProfiler | Bioconductor website |  |
| R package: ggplot2 | Bioconductor website |  |
| R package: ggstatplot | Bioconductor website |  |
| R package: ggpur | Bioconductor website |  |
| R package: pheatmap v1.0.2 | Bioconductor website |  |
